# The peptide EPFL8 represses embryonic stomatal precursor formation independently of TOO MANY MOUTHS in Arabidopsis

**DOI:** 10.1093/plphys/kiaf487

**Published:** 2025-10-06

**Authors:** Qin He, Aditya Birla, Guillaume Lamoureux, Huiliang Zhang, Xingyun Qi

**Affiliations:** Department of Biology, Rutgers University, Camden, NJ 08103, USA; Center for Computational and Integrative Biology, Rutgers University, Camden, NJ 08103, USA; Center for Computational and Integrative Biology, Rutgers University, Camden, NJ 08103, USA; Center for Computational and Integrative Biology, Rutgers University, Camden, NJ 08103, USA; Department of Chemistry, Rutgers University, Camden, NJ 08103, USA; Department of Medical Genetics and Molecular Biochemistry, Lewis Katz School of Medicine, Temple University, Philadelphia, PA 19140, USA; Department of Biology, Rutgers University, Camden, NJ 08103, USA; Center for Computational and Integrative Biology, Rutgers University, Camden, NJ 08103, USA

## Abstract

Plants regulate gas exchange through stomata, which are specialized valves on the aerial epidermis. Stomatal development involves tightly controlled transitions orchestrated by basic helix-loop-helix transcription factors, including SPEECHLESS (SPCH). These factors coordinate cell division and differentiation during stomatal development. Recent studies have underscored the importance of cell–cell communication in this process, mediated by secreted peptide ligands of the EPIDERMAL PATTERNING FACTOR (EPF) family and a Leucine-Rich-Repeat Receptor complex that includes ERECTA family receptor kinases and the receptor TOO MANY MOUTHS (TMM). Although mature stomata do not form during embryogenesis, stomatal cell fate is determined at these early stages. In this study, we discovered that *EPFL8* is highly expressed during early embryogenesis and inhibits stomatal development by negatively regulating SPCH. Furthermore, *TMM*, a SPCH target, interferes with EPFL8-mediated signaling. In the absence of TMM, *EPFL8* expression extends into later embryogenesis, which aligns with the reduced number of stomatal precursors observed in *tmm* mutants. These findings reveal that EPFL8 functions as an embryonic peptide ligand that suppresses stomatal formation via SPCH regulation, highlighting the diversification of EPF family members in fine-tuning plant epidermal patterning.

## Teaser

Embryonic peptide signaling that represses the transcription factor SPCH is attenuated by SPCH-induced receptor, thereby fine-tuning stomatal prepatterning.

## Introduction

Stomata are micropores found on the aerial surface of most land plants (Ruszala et al. 2011; Duckett and Pressel 2018), playing a crucial role in mediating gas exchange between the plant's inner tissues and the surrounding environment. They facilitate 2 key physiological processes, photosynthesis and transpiration (Becklin et al. 2021; Han et al. 2021). In the model dicot plant *Arabidopsis thaliana*, stomatal development follows a traceable process that begins with a precursor cell known as the meristemoid mother cell (MMC) (Han and Torii 2016). The MMC undergoes 1 to 4 rounds of asymmetric division, also referred to as amplifying divisions, positioning a small meristemoid cell in the center and several large neighboring cells called stomatal lineage ground cells. The meristemoid then differentiates into a guard mother cell (GMC), which divides symmetrically once to form a pair of guard cells (GCs) that enclose the stomatal pore.

The molecular pathway regulating stomatal development was identified in the last 2 decades, and it involves a complex network of signaling components. From upstream to downstream, the pathway includes secreted peptides from the EPIDERMAL PATTERNING FACTOR (EPF) family, the Leucine-Rich-Repeat (LRR) receptor complex, the MAPK cascades, and several basic-Helix-Loop-Helix (bHLH) transcription factors. Within the EPF family, members such as EPF1, EPF2, and EPFL4/5/6 act as inhibitors of stomatal development (Hara et al. 2007, 2009; Hunt and Gray 2009; Abrash and Bergmann 2010; Abrash et al. 2011), while EPFL9/STOMAGEN promotes stomatal formation (Kondo et al. 2010; Sugano et al. 2010). The EPF ligands are recognized by a receptor complex (Lee et al. 2012, 2015; Lin et al. 2017), which includes receptor kinases in the ERECTA (ER) family (Shpak et al. 2005) and the SOMATIC EMBRYOGENESIS RECEPTOR KINASE (SERK) family (Meng et al. 2015). A key modulator of the EPF signaling is the receptor protein TOO MANY MOUTHS (TMM), where some EPF ligands require TMM for signaling, while some others do not (Yang and Sack 1995; Nadeau and Sack 2002). The inhibitory EPF2 signal transmitted through this receptor complex activates the MAPK cascade (Bergmann et al. 2004; Wang et al. 2007; Lampard et al. 2009; Lee et al. 2015), leading to the phosphorylation and degradation of the bHLH transcription factor SPEECHLESS (SPCH) (Lampard et al. 2008). SPCH, along with its 2 homologous transcription factors, MUTE and FAMA, controls the 3 major steps of stomatal development, the asymmetric cell divisions (ACDs) of the meristemoids, the differentiation of the late meristemoid into the GMC, and the symmetric division and subsequent differentiation of the GCs (Ohashi-Ito and Bergmann 2006; MacAlister et al. 2007; Pillitteri et al. 2007). Additionally, a broadly expressed bHLH transcription factor family, including SCREAM (SCRM)/INDUCER of CBF EXPRESSION 1 (ICE1) and SCRM2, partners with SPCH, MUTE, and FAMA to coordinate the entire process of stomatal development (Kanaoka et al. 2008).

While stomata facilitate gas exchange in the postembryonic sporophyte following seed germination, no stomata are formed during seed development (Peterson and Torii 2012; Smit et al. 2023; Geisler and Sack 2002; Danzer et al. 2015; Smit et al. 2023). Seeds develop through embryonic growth and endosperm proliferation. After fertilization, the zygote cell undergoes active divisions, progressing through several stages, including the 2-cell, 8-cell, 32-cell, globular, triangular, heart, torpedo, upright, and walking-stick stages, ultimately forming a mature embryo (Goldberg et al. 1994; Jurgens 2001). Once the embryo reaches maturity, cell divisions are halted, and storage compounds begin to accumulate. During late embryogenesis, a dehydration process occurs, causing the seed to lose up to 90% of its water. This results in seed dormancy, a state in which the seed becomes “quiescent” until conditions are favorable for germination.

Although mature stomata are absent in embryos (Peterson and Torii 2012; Smit et al. 2023), several studies have reported the presence of stomatal precursors and stomatal-lineage regulators during embryogenesis (Geisler and Sack 2002; Danzer et al. 2015). Smit et al. conducted a comprehensive analysis of stomatal precursor prepatterning in *Arabidopsis* embryos (Smit et al. 2023). They demonstrated that SPCH protein first appeared at the heart stage, and one round of ACD only started to occur at the torpedo stage. Furthermore, early ectopic expression of SPCH under the *ARABIDOPSIS THALIANA MERISTEM LAYER 1 (ATML1)* promoter failed to accelerate embryonic ACD before the late torpedo stage. Additionally, the authors observed fewer cells in the protodermal patterns of mature embryos in imbibed seeds in *tmm* mutants—an unexpected phenotype not accounted for by their proposed model. This discrepancy suggests the presence of unknown regulators in the stomatal regulatory network during embryonic pattern formation.

In this study, we identified *EPFL8*, an early embryonic gene that inhibits stomatal development by targeting SPCH protein during the early stages of embryogenesis. Notably, *EPFL8* expression is upregulated in *tmm* mutants, and its signaling pathway functions independently of TMM, offering an explanation for the embryonic stomatal precursor phenotype observed in *tmm* mutants.

## Results

### TMM promotes stomatal precursor formation during embryogenesis

**Figure 1.**
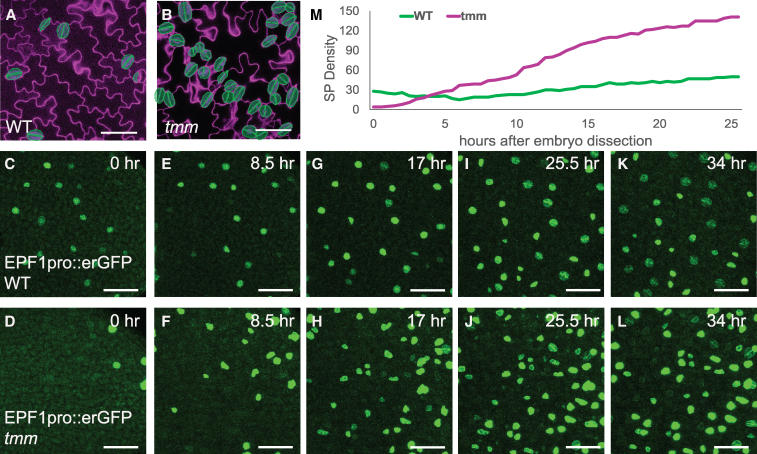
More stomata are formed after germination in *tmm* mutants. **A**, **B)** Confocal images showing the abaxial side of cotyledons of 5 day-postgermination (dpg) WT plants (A) and *tmm* mutants (B). Mature stomata are highlighted in green. *N* = 6 for both WT and *tmm* images, scale bar: 20 µm. **C** to **L)** Time-lapse confocal images showing the abaxial side of cotyledons in 1 dpg WT plants (C, E, G, I, K) and *tmm* mutants (D, F, H, J, L) expressing the *EPF1pro::erGFP* marker, which labels late meristemoids and newly formed stomata. The corresponding hours after germination are indicated in each panel. Scale bar: 50 µm. **M)** Quantification analysis of cells expressing *EPF1pro::erGFP* in WT plants and *tmm* mutants over a 34-h period postgermination. SP density: stomata and precursor density.

TMM is a receptor-like protein known to mediate the inhibitory signals of several EPF ligands, thereby playing a critical role in defining meristemoid cell fate during stomatal development (Geisler et al. 1998; Nadeau and Sack 2002; Lee et al. 2012; Qi et al. 2017). Mutations in TMM result in severe stomatal clustering on cotyledons after germination (Fig. 1, A and B). In imbibed seeds, no mature stomata formed. Analyzing cell shapes, neighbor orientations, and the overall cell pattern of the embryonic epidermis, Smit et al. observed fewer cells in *tmm* embryos compared to wild-type (WT) embryos (Smit et al. 2023). To verify whether these ACD events correspond to stomatal precursor formation, we employed a stomatal-lineage-specific marker protein *EPF1pro::erGFP* (endoplasmic reticulum GFP) to label late meristemoids both before and after germination (Hara et al. 2007; Qi et al. 2017). As shown in Fig. 1, C and D, fewer *EPF1pro::erGFP*-positive cells were detected in imbibed *tmm* mutant seeds compared to those in WT seeds, confirming fewer meristemoids and an inhibitory stomatal phenotype in *tmm* seeds before germination.

To further verify the differing stomatal phenotype in *tmm* mutants before and after germination, we monitored stomatal development over a 34-h period in dissected imbibed seeds (Fig. 1, C to L, Videos 1 and 2). Our results showed that although *tmm* mutants began with fewer stomatal precursor cells at germination, they rapidly caught up to WT levels within 4 h (Fig. 1M) and eventually surpassed them. In contrast, WT plants, which started with more than twice the number of stomatal precursor cells, developed at a slower, steady rate, resulting in a scattered stomatal pattern (Fig. 1). Consistent with previous reports (Smit et al. 2023), the first batch of stomata formed via direct symmetric division of embryonic stomatal precursors between 13 and 15 h after dissection. These findings indicate that the absence of TMM inhibits stomatal prepatterning during embryogenesis while promoting rapid stomatal development postgermination.

**Figure 2.**
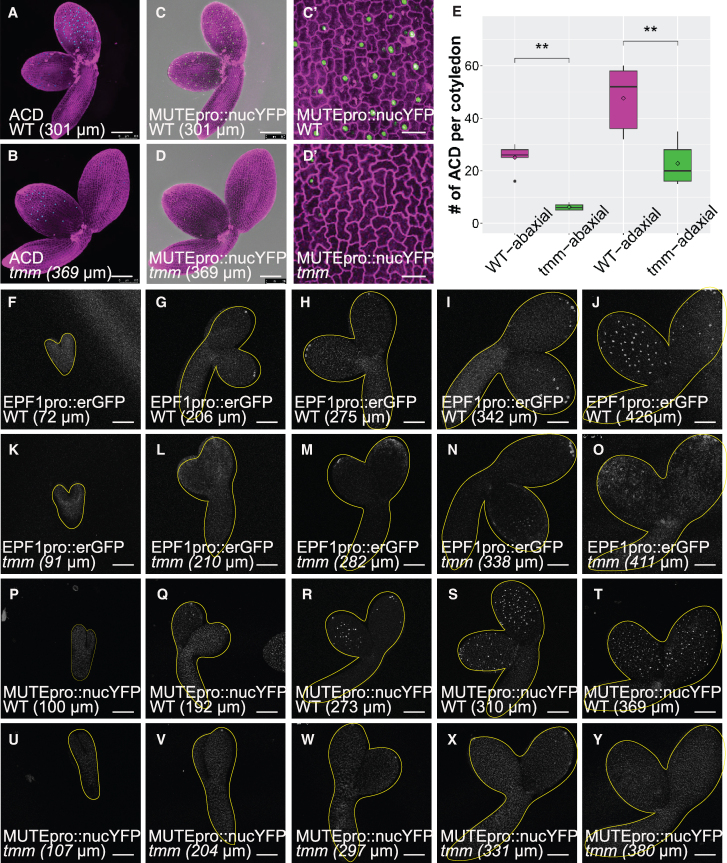
Fewer stomatal precursors are formed during embryogenesis in *tmm* mutants. **A** to **D′)** Propidium iodide (PI)-stained images of WT plants (A, C, C′) and *tmm* mutants (B, D, D′) with ACD events labeled on both the abaxial side and adaxial side (A, B) or expressing *MUTEpro::nucYFP* (C, C′ D, D′). *N* = 6 for both WT and *tmm* images. Scale bar: 100 µm (A to D); 20 µm (C′, D′). **E)** Boxplot showing quantitative analysis of ACD events on both leaf sides in WT plants and *tmm* mutants. Statistical analysis was performed using Student’s *t*-test. **P < 0.01. *N* = 5 for all 4 samples. Center line: Represents the median (Q2), which is the 50th percentile of the data. Box edges: Define the interquartile range (IQR), with the lower edge being the first quartile (Q1, the 25th percentile) and the upper edge being the third quartile (Q3, the 75th percentile). The box contains the middle 50% of the data. Whiskers: Extend from the box to the minimum and maximum data points that are not outliers (value that is more than 1.5×IQR below Q1 or above Q3). Small internal diamond: indicates the mean (average) value. Points (dots outside the whiskers): Outliers. **F** to **O)** Confocal images showing *EPF1pro::erGFP* labeled stomatal precursors at different stages during embryogenesis in WT plants (F to J) and *tmm* mutants (K to O). **P** to **Y)** Confocal images showing *MUTEpro::nucGFP*-labeled stomatal precursors at different stages during embryogenesis in WT plants (P to T) and *tmm* mutants (U to Y). Confocal images from Fto Y display the GFP or YFP signal, represented in white. The lengths of the abaxial side of cotyledons are indicated in each panel. Scale bar: 100 µm. *N* = 6 for all images.

ACD events are essential for stomatal precursor formation. Smit et al. defined these events based on cell shape, neighbor orientation, and overall embryonic cell pattern, and demonstrated that only one round of ACD occurs during stomatal precursor formation in embryogenesis (Smit et al. 2023). To investigate the correlation between stomatal precursor formation and ACD events, we applied the same criteria to analyze cotyledons from *MUTEpro::nucYFP (nucleus-localized YFP)* reporter lines, which specifically mark meristemoids undergoing their final ACD (Pillitteri et al. 2007; Han et al. 2018). As shown in Fig. 2, A, B, and E, the number of ACD events (highlighted by dark blue dots on the abaxial side and light blue dots on the adaxial side) was significantly reduced in *tmm* cotyledons compared to WT. Furthermore, the strong correlation between YFP-positive cells and ACD events (Fig. 2, C, D, C′, D′) confirms a dramatic reduction in stomatal precursors in *tmm* embryos.

To track stomatal precursor dynamics during embryogenesis, we monitored *EPF1pro::erGFP* expression at various embryonic developmental stages (Fig. 2). Embryos were dissected from the 4th to the 8th siliques and categorized based on their morphology. Additionally, the lengths of the emerging cotyledons were measured to compare developmental stages, following the protocol described by Smit et al. (2023). In WT embryos, *EPF1* promoter activity was not detected at the torpedo stage (Fig. 2F). At the upright stage, a bright GFP signal appeared at the cotyledon tips on both sides (Fig. 2G). As the embryo developed, more cells on both adaxial and abaxial sides expressed *EPF1pro::erGFP*, eventually displaying a well-scattered pattern (Fig. 2, H to J). Starting from the late upright stage, the adaxial side bore more GFP-positive cells than the abaxial side (Fig. 2, I and J), suggesting leaf polarity also affects stomatal development.

In *tmm* mutants, *EPF1pro::erGFP* expression was not detected at the torpedo stage (Fig. 2K), and was restricted to the cotyledon tips at the upright stage and remained largely unchanged until the walking-stick stage (Fig. 2, L to N). Even in mature embryos, GFP-positive cells were limited to the abaxial tip region, with a few detected on the adaxial side (Fig. 2O). This phenotype was further validated using the *MUTEpro::nucYFP* reporter, which reproduced similar patterns in both WT and *tmm* embryos (Fig. 2, P to Y). These findings confirmed that fewer cells enter the stomatal lineage in *tmm* embryos, suggesting an enhanced inhibitory signal of stomatal development when TMM is absent.

Our data suggest that TMM promotes stomatal precursor formation during embryogenesis, with differential effects on the adaxial and abaxial cotyledon sides. This role is critical for maintaining proper stomatal prepatterning before germination. In its absence, the inhibitory signal predominates, reducing the number of stomatal precursors during embryogenesis while allowing rapid, unregulated stomatal development postgermination.

### Early *EPFL8* expression persists into later embryonic stages in *tmm* mutants, and its signaling inhibits stomatal formation independently of TMM

TMM is a receptor protein that binds to ER family members to mediate EPF signaling (Lee et al. 2012; Lin et al. 2017). Unlike ER family members, which are essential for EPF ligand perception (Shpak et al. 2005), TMM acts as a modulator in signal transduction (Lin et al. 2017). Certain EPF family members, such as EPF1, EPF2, and EPFL9, require TMM for effective signal transduction (Hara et al. 2007; Hunt and Gray 2009; Lee et al. 2012). In contrast, the EPFL4/5/6 subfamily inhibits stomatal formation without relying on TMM (Abrash and Bergmann 2010; Abrash et al. 2011). Interestingly, the stomatal inhibitory effects of EPFL4/5/6 are enhanced in the absence of TMM (Supplementary Fig. S1) (Abrash et al. 2011), suggesting that TMM impairs their signal transduction.

**Figure 3.**
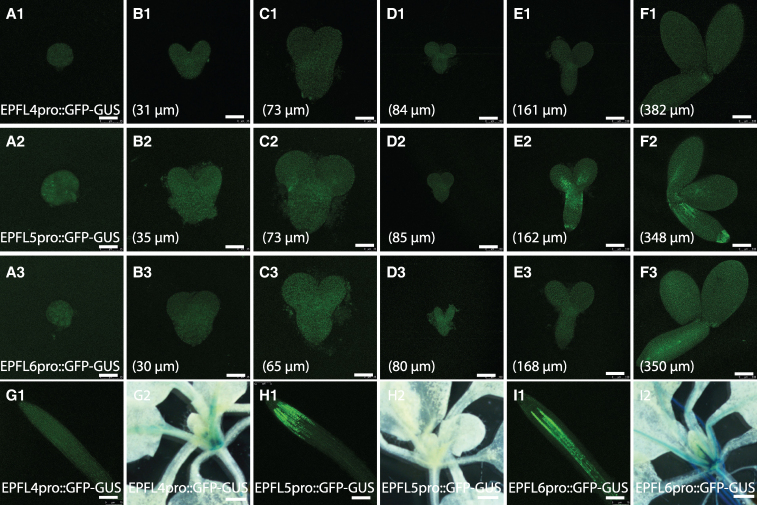
The EPF4/5/6 members show differential expression pattern during embryogenesis. Images of multiple stages of embryogenesis in plants expressing various reporter constructs are shown: *EPFL4pro::EGFP-GUS* **(A1** to **F1)**, *EPFL5pro::EGFP-GUS* **(A2** to **F2)**, *EPFL6pro::EGFP-GUS* **(A3** to **F3)**. Confocal images from A1 to F3 display the EGFP signal. The lengths of the abaxial side of cotyledons are indicated in each panel. Scale bars: 25 µm **(A1** to **B1**, **A2** to **B2**, **A3** to **B3)**; 100 µm **(C1** to **F1**, **C2** to **F2**, **C3** to **F3)**. *N* = 6 for all images. Postgermination root tip images **(G1**, **H1**, **I1)** and shoot apical meristem images **(G2**, **H2**, **I2)** show the GFP signal or GUS signal from *EPFL4pro::EGFP-GUS* **(G1, G2),** *EPFL5pro::EGFP-GUS* **(H1, H2)**, and *EPFL6pro::EGFP-GUS* **(I1, I2)**. The lengths of the abaxial side of cotyledons are indicated in **A1** to **F3**. Scale bars: 25 µm **(A1** to **B1**, **A2** to **B2**, **A3** to **B3)**, 100 µm **(C1** to **F1**, **C2** to **F2**, **C3** to **F3)**, 75 µm **(G1, H1, I1)**, 2 mm **(G2, H2, I2)**. *N* = 6 for all images.

**Figure 4.**
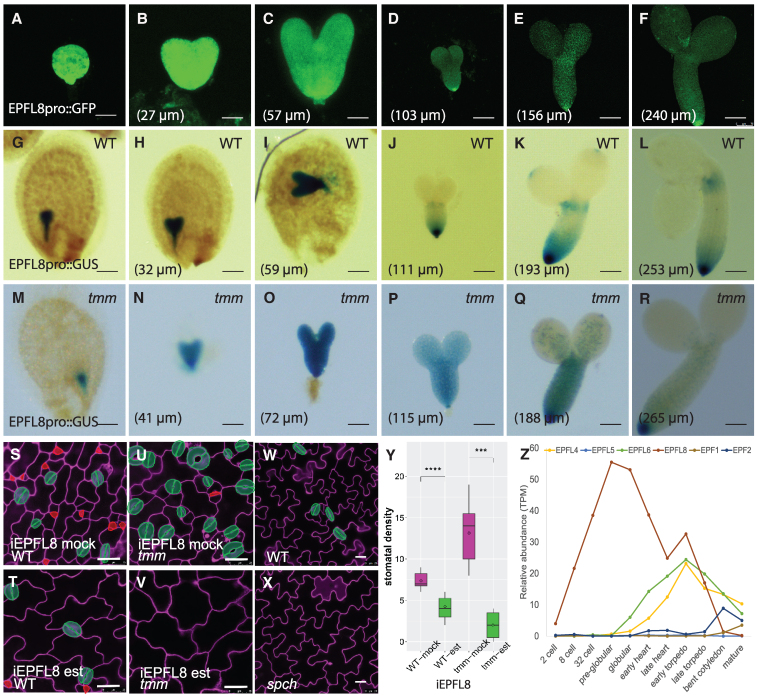
The early embryonic EPFL8 inhibits stomatal formation independent of TMM. **A**to **R)** Images of multiple stages of embryogenesis in plants expressing *EPFL8* transcriptional reporters are shown: *EPFL8pro::GFP* (A to F), *EPFL8pro::GUS* in WT embryos (G to L) or in *tmm* embryos (M to R). Confocal images from A to F display the EGFP signal, represented in green, while images from G to R display the GUS signal driven by the *EPFL8* promoter, shown in blue. The lengths of the abaxial side of cotyledons are indicated in each panel. Scale bars: 25 µm (A and B); 100 µm (C to R). *N* = 6 for all images. **S** to **V)** Stomatal phenotype of the abaxial side of cotyledons in *iEPFL8* plants in the WT background (S, T) or the *tmm* mutant background (U, V), treated with either mock (S, U) or estradiol (T, V). *iEPFL8*: inducible *EPFL8*; Est: estradiol induction. **W** to **X)** Stomatal phenotype of WT plants (W) and *spch* mutants (X). Mature stomata are highlighted in green. Scale bars: 25 µm for all panels. *N* = 6 for all images. **Y)** Boxplot showing quantification of stomatal density in transgenic *iEPFL8* plants in WT and *tmm* backgrounds treated with mock or estradiol. Statistical analysis was performed using Student's *t*-test. ***P < 0.001, ****P < 0.0001. *N* = 8, 12, 7, 7 for WT-mock, WT-est, tmm-mock, tmm-est, respectively. Center Line: Represents the Median (Q2), which is the 50th percentile of the data. Box Edges: Define the IQR, with the lower edge being the first quartile (Q1, the 25th percentile) and the upper edge being the third quartile (Q3, the 75th percentile). The box contains the middle 50% of the data. Whiskers: Extend from the box to the minimum and maximum data points that are not outliers (value that is more than 1.5×IQR below Q1 or above Q3). Small internal diamond: indicates the mean (average) value. Points (dots outside the whiskers): Outliers. **Z)** Transcript abundance of stomatal genes as reported in Hofmann et al. (2019). *N* = 3 according to Hofmann et al. (2019). TPM: transcript per million.

To determine whether EPFL4/5/6 are responsible for inhibiting stomatal precursors in *tmm* embryos, we analyzed their expression patterns during embryogenesis using the established transcriptional reporter lines: *EPFL4pro::EGFP-GUS*, *EPFL5pro::EGFP-GUS*, and *EPFL6pro::EGFP-GUS* (Kosentka et al. 2019), all of which showed expected postgermination expression in root tips and shoot apical meristems (Fig. 3, G1 to I2). As shown in Fig. 3, limited *EPFL4* expression was detectable starting from the upright stage (Fig. 3, A1 to F1). *EPFL5* expression was absent until late embryogenesis, where it was observed in the future root tip, the upper hypocotyl, and the basal part of the cotyledons (Fig. 3, A2 to F2). *EPFL6* expression was restricted to certain inner cells of the hypocotyl in mature embryos (Fig. 3, A3 to F3), consistent with previous findings (Kosentka et al. 2019). Additionally, *EPFL6* has been reported to localize in the chalaza end rather than the embryo itself (Abrash and Bergmann 2010). The RNA-seq data from Hofmann et al. (2019) detected *EPFL4* and *EPFL6* expression during embryogenesis, with modest levels observed at early stages (Fig. 4Z). Our GFP data best indicate that *EPFL4* and *EPFL6* were absent on the epidermis, but our approach does have limitations in detecting GFP signals from interior tissues, which leads to the differences between these 2 approaches. Since the stomatal precursor phenotype in *tmm* mutants emerges on the embryonic epidermis at early stages, it is likely that, besides the limited contribution from EPFL4 and EPFL6, other EPF ligands contribute to stomatal prepatterning inhibition during embryogenesis.

To test this hypothesis, we performed a phylogenetic analysis using the full-length peptide sequences of the 11 EPF family members in *Arabidopsis* and found that EPFL8 is a close homologue of the subfamily as EPFL4/5/6 (Supplementary Fig. S2A), consistent with Takata's analysis (Takata et al. 2013). Although *EPFL8* expression is undetectable after germination (Supplementary Fig. S3A) (Kosentka et al. 2019), our RT-qPCR result (Supplementary Fig. S3B) and the RNA-seq data from Hofmann et al. (2019) detected high expression levels of *EPFL8* during embryogenesis (Fig. 4Z). To investigate the spatiotemporal expression pattern of *EPFL8*, we generated a transcriptional reporter line, *EPFL8pro::GFP*. As expected, a strong GFP signal was observed at multiple stages of embryogenesis (Fig. 4, A to F). Since GFP can diffuse between cells, particularly during early embryogenesis (Stadler et al. 2005), we also developed an *EPFL8pro::GUS* reporter line (Fig. 4, G to L). Both reporters demonstrated that *EPFL8* is strongly expressed during early embryogenesis, including the globular, heart, and torpedo stages. As embryogenesis progressed, the *EPFL8* signal weakened in the cotyledon region but remained prominent in the hypocotyl, particularly in the future root tip region.

To investigate the effect of *TMM* on *EPFL8*, we examined both its expression pattern and function in *tmm* mutants. Up to the late torpedo stage, *EPFL8* showed a strong signal throughout the *tmm* embryos (Fig. 4, M to O), similar to WT. As embryogenesis progressed, the overall *EPFL8* signal gradually weakened (Fig. 4, P to R). Unexpectedly, while variability in *EPFL8* expression intensity was observed among the samples at each stage, in many later-stage *tmm* embryos—particularly in the cotyledons—*EPFL8* expression remained high (Fig. 4, P to R), in contrast to the fading signal in WT cotyledons. This finding is consistent with our RT-qPCR results (Supplementary Fig. S3C), which showed higher *EPFL8* expression in the 5th and 6th *tmm* siliques compared to WT, while the 3rd and 4th siliques exhibited comparable expression between the 2 backgrounds.

To assess whether EPFL8 inhibits stomatal formation and if its signal transduction requires TMM, we generated estradiol-inducible *EPFL8* lines. Since stomatal development is incomplete during embryogenesis, we evaluated EPFL8 function in young cotyledons during early germination. Upon estradiol induction, *EPFL8* expression increased by over 300-fold in both WT seedlings (324.3-fold) and *tmm* mutant seedlings (323.8-fold) (Supplementary Fig. S3D). In WT plants, elevated *EPFL8* expression led to a slight reduction in the stomatal index (Fig. 4, S and T). Under normal conditions, *tmm* mutants exhibited a higher stomatal index on the abaxial side of cotyledons (Fig. 4U). However, when estradiol was applied to induce *EPFL8* expression, nearly no stomata formed on the abaxial side of *tmm* cotyledons (Fig. 4V). Additionally, the cotyledon epidermis of induced *EPFL8* plants primarily consisted of large pavement cells, resembling the lack of ACD phenotype in *spch* mutants (Fig. 4X) (MacAlister et al. 2007). These results indicate that EPFL8 is a potent inhibitor of stomatal development independent of TMM, with its effect most likely exerted at the ACD step.

### *EPFL8* co-exists with *ER* prior to the appearance of *TMM*

**Figure 5.**
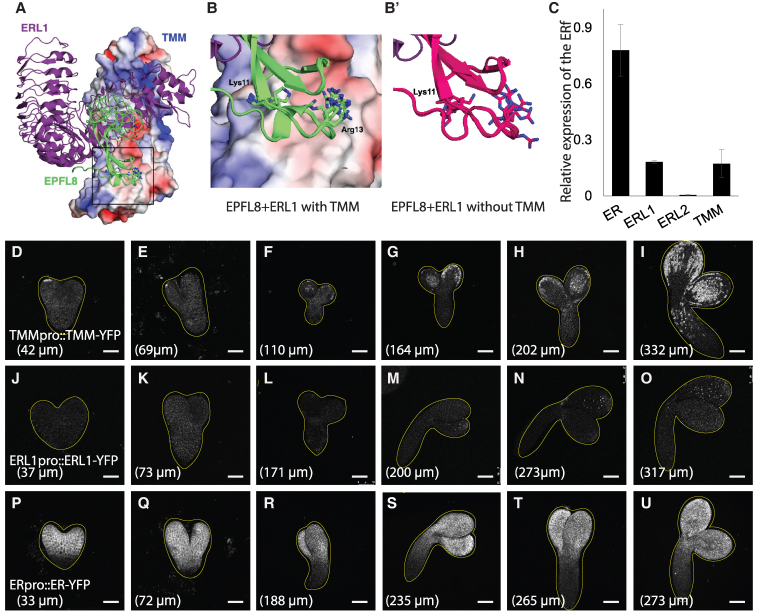
The spatiotemporal expression pattern of the receptors differs during embryogenesis. **A)** Homology model of the ERL1-TMM-EPFL8 complex. ERL1 is shown as purple ribbons, EPFL8 as green ribbons (10 structural models), and TMM as an electrostatic potential surface (negative in red, positive in blue). **B)** Enlarged view of the black-framed region from (A), illustrating steric and electrostatic constraints by TMM on EPFL8 residues Lys11 and Arg13. (B′) Homology model of the ERL1-EPFL8 complex without TMM, showing EPFL8 as magenta ribbons. **C)** RT-qPCR results showing relative expression levels of *ER*, *ERL1*, *ERL2*, and *TMM* in the 6th silique. Data Normalized to actin expression. Error bars indicated standard error. *N* = 3. **D** to **U)** Confocal images of embryogenesis stages in plants expressing *TMMpro::TMM-YFP* (D to I), *ERL1pro::ERL1-YFP* (J to O), and *ERpro::ER-YFP* (P to U). Confocal images from D to U display the YFP signal, represented in white. The lengths of the abaxial side of cotyledons are indicated in each panel. Scale bar: 50 µm (D, E, J, K, P, Q), 100 µm (F to I, L to O, R to U). *N* = 6 for all images.

Our phylogenetic analysis, biochemical evidence (Lin et al. 2017), and our genetic results indicate that EPFL8 does not require TMM for signal transduction. In fact, the presence of TMM impairs the EPFL8 signal (Fig. 4, S to V, and Y), consistent with the behavior of the EPFL4/5/6 subfamily (Abrash et al. 2011). Lin et al. resolved the crystal structures of EPF ligands and their receptor complexes—including both the EPF1/2 and EPFL4/5/6 subfamilies—and demonstrated that TMM acts as a specificity modulator for the binding of these subfamilies to ER family receptors (Lin et al. 2017). To further investigate how TMM hinders EPFL8 signal transduction, we leveraged the available protein structures to 10 homology models of the EPFL8-ERL1-TMM complex (Fig. 5, A and B). For comparison, we also generated 10 structural models of the EPFL8-ERL1 complex without TMM (Fig. 5B′). The homology models suggest that the charged residue EPFL8_Arg13_ (a glycine in EPF1) can be accommodated by TMM when bound to ERL1. However, the charged residue EPFL8_Lys11_ (an alanine in EPF1) is sterically and electrostatically hindered by the presence of TMM (Supplementary Fig. S2, B and C). Specifically, TMM creates a steric constraint on residue Lys11, reducing its conformational flexibility and buries its ammonium moiety into an electropositive region of the TMM surface (Fig. 5B). This environment provides no opportunities for favorable electrostatic interactions, making it unlikely for EPFL8 to bind a preformed ERL1-TMM complex.

In the absence of TMM, residue Lys11 remains exposed to the solvent and loses minimal conformational entropy upon binding with ERL1. However, when TMM is present, Lys11 loses more conformational entropy. This loss of entropy is not compensated by favorable electrostatic interactions with TMM, as TMM lacks negatively charged groups in the vicinity of (positively charged) Lys11. Consequently, the homology models suggest that TMM has an overall destabilizing effect on the ERL1-EPFL8 interaction or, equivalently, competes with EPFL8 for ERL1 binding.

To investigate whether *TMM* co-expresses with *EPFL8*, we examined embryos expressing a functional *TMMpro::TMM-YFP* cassette during embryogenesis. The TMM-YFP signal first appeared after the heart stage, localized at the cotyledon tip (Fig. 5D). As the embryo development proceeded, *TMM* expression gradually expanded to both sides of the cotyledons and into the hypocotyl tip (Fig. 5, E to I), consistent with Smit and colleagues’ findings (Smit et al. 2023). Notably, strong *EPFL8* expression was detected earlier than *TMM* (Figs. 4 and 5), creating a spatiotemporal environment conducive to efficient EPFL8 signaling before TMM becomes prominent.

The ER family members form a complex with TMM to perceive several EPF ligands identified so far (Abrash et al. 2011; Lee et al. 2012; Sanchez-Rodriguez et al. 2018). While our findings indicate that the EPFL8 ligand does not require TMM, the role of the ER family in EPFL8 signal transduction remains unclear. Previous studies have reported that ER family members can physically bind EPFL8 independently of TMM (Lin et al. 2017). Given the distinct transcriptional profiles of plants before and after germination, we first examined the expression patterns of the 3 ER members during embryogenesis. Our RT-qPCR analysis showed minimal expression of *ERL2* in silique #6 (Fig. 5C). In contrast, both *ER* and *ERL1* were expressed, with *ER* exhibiting higher expression levels than *ERL1* (Fig. 5C). To determine whether *ER* or *ERL1* spatiotemporally coexists with *EPFL8* during embryogenesis, we analyzed embryos from transgenic plants bearing *ERpro::ER-YFP* or *ERL1pro::ERL1-YFP* reporters (Fig. 5, J to U). Consistent with the RT-qPCR results, high *ER* expression was observed in the protodermal cells across the entire cotyledon throughout embryogenesis (Fig. 5, P to U). In contrast, *ERL1* expression was detected only after the torpedo stage (Fig. 5, J to O). Even in nearly mature embryos, *ERL1* was mostly restricted to the adaxial side of the cotyledon (Fig. 5O). These findings suggest that *ER* expression strongly overlaps with *EPFL8* in cotyledons at the early stages of embryogenesis, making it a likely receptor kinase for perceiving the EPFL8 signal.

### The EPFL8 signal post-translationally targets the SPCH protein

The expression patterns of *EPFL8*, *TMM*, and *ER* suggest that a strong EPFL8 signal is transmitted before *TMM* expression begins. Notably, enhanced EPFL8 signaling results in an epidermis composed predominantly of large cells, a loss of ACD phenotype similar to that observed in *spch* mutants (Fig. 4, W and X) and the *scrm scrm2* double mutants (Kanaoka et al. 2008). SCRM/SCRM2 heterodimerizes with SPCH to promote the transcription of stomatal lineage-related genes, including *TMM* and *EPF2* (Kanaoka et al. 2008; Lau et al. 2014; Horst et al. 2015). During embryogenesis, *SPCH* and *SCRM* are first detected at the heart stage; however, ACD events do not occur until the torpedo stage (Smit et al. 2023). Even when *SPCH* and *SCRM* are expressed precociously in early embryos, ACDs are not induced before the torpedo stage (Smit et al. 2023). Notably, *SPCH* is detected in only a subset of protodermal cells, whereas *SCRM* protein is broadly present in both protodermal and internal tissues (Smit et al. 2023). Although ectopic *SPCH* expression can induce *TMM* and *EPF2*, overactivation of *SCRM* has no effect on *EPF2* expression (Smit et al. 2023). These findings suggest that early SPCH is regulated at the post-transcriptional or post-translational level, resulting in the absence of ACD during early embryogenesis (Peterson and Torii 2012; Smit et al. 2023). We hypothesize that, in the absence of TMM, EPFL8 signaling mediates, at least in part, the negative regulation of SPCH protein.

**Figure 6.**
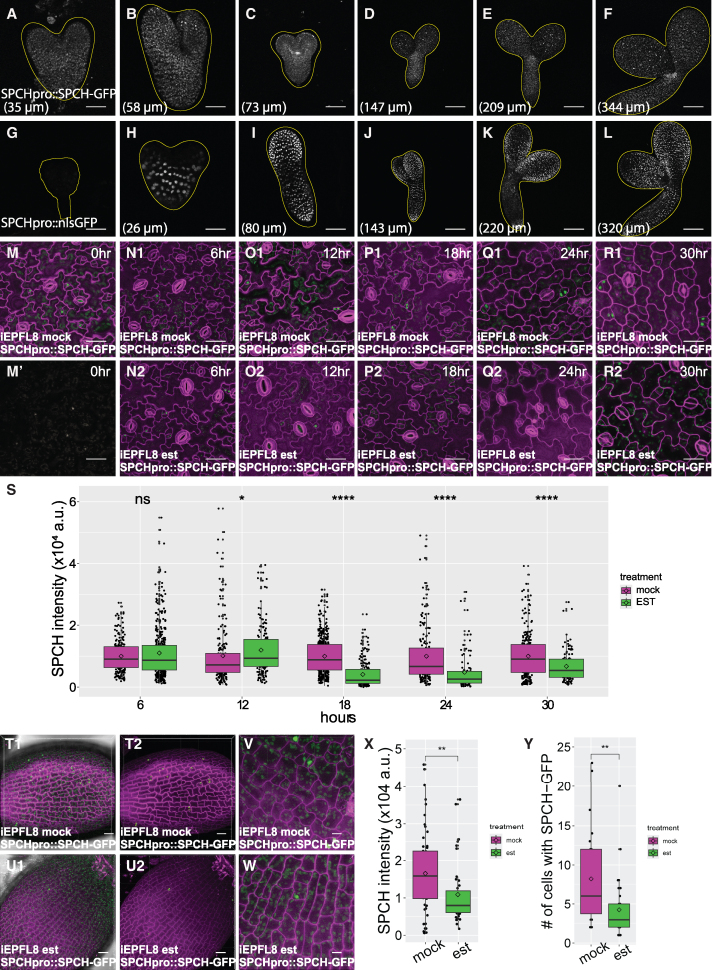
The EPFL8 signal negatively regulates SPCH protein abundance during embryogenesis. **A** to **L)** Confocal images depicting embryogenesis stages in plants expressing *SPCHpro::SPCH-GFP* (A to F) and *SPCHpro::nlsGFP* (G to L). *SPCHpro::SPCH-GFP:* SPCH translational reporter showing SPCH-GFP in the nucleus; *SPCHpro::nlsGFP*: *SPCH* transcriptional reporter showing nucleus-localized GFP signal. Confocal images from A to L display the GFP or YFP signal, represented in white. The lengths of the abaxial side of cotyledons are indicated in each panel. Scale bar: 25 µm (A, B, G, H), 50 µm **C, I)**, 100 µm (D to F, J to L). *N* = 6 for all images. (M-R2, T1-W) Confocal images of 4 dpg cotyledon (M-R2) or embryos in #8 silique (T1-W) of *iEPFL8* plants expressing *SPCHpro::SPCH-GFP* treated with mock (M, M′, N1, O1, P1, Q1, R1, T1, T1, V) or estradiol (N2, O2, P2, Q2, R2, U1, U2, W). The induction time points were labeled in hours in M-R2. T2 and U2 are the Imaris surface reconstruction of GFP signal in T1 and U1, respectively. T1-U2 showed the entire cotyledon, while V and W displayed enlarged views. Cell outlines are marked with PI staining. Scale bar: 50 µm (M-R2), 30 µm (T1-U2), 10 µm (V, W). **S**, **X**) Boxplot and Dotplot showing quantification of SPCH intensity per nucleus in transgenic *iEPFL8* embryos (Q) or cotyledons (Y) bearing *SPCHpro::SPCH-GFP*, treated with mock or estradiol at different time points. Intensity normalized to mock treatment averages in (Y). The Student’s *t*-test was used for statistical analysis. ****P < 0.0001. *n* = 53, 44 (embryo mock, est) *n* = 226, 213, 437, 195, 257 (cotyledon mock 6, 12, 18, 24, 30 h); *n* = 422, 179, 182, 100, 109 (cotyledon est 6, 12, 18, 24, 30 h). **Y)** Boxplot and Dotplot showing quantification of the number of cells expressing *SPCH-GFP* per embryo in #8 silique of transgenic *iEPFL8 SPCHpro::SPCH-GFP* plants, treated with mock or estradiol. The Student’s *t*-test was used for statistical analysis. **P < 0.01. *n* = 24 (mock), 39 (est). For elements in all boxplots, center line: Represents the median (Q2), which is the 50th percentile of the data. Box edges: Define the IQR, with the lower edge being the first quartile (Q1, the 25th percentile) and the upper edge being the third quartile (Q3, the 75th percentile). The box contains the middle 50% of the data. Whiskers: Extend from the box to the minimum and maximum data points that are not outliers (value that is more than 1.5×IQR below Q1 or above Q3). Small internal diamond: indicates the mean (average) value. Points (dots outside the whiskers): Outliers.

To test this hypothesis, we examined the SPCH protein during embryogenesis using a functional translational reporter, *SPCHpro::-SPCH-GFP* (Horst et al. 2015), in *spch* background. No GFP signal was detected at the early heart stage (Fig. 6A). SPCH protein was first observed at the tip region of the cotyledon during the early torpedo stage (Fig. 6B), gradually spreading across the cotyledon in a scattered pattern on both sides (Fig. 6, C to F). These SPCH-positive cells displayed a distribution pattern similar to that of cells expressing *TMMpro:: TMM-YFP* (Fig. 5, D to I), supporting the notion that SPCH promotes *TMM* transcription and aligning with previous findings that ectopic *SPCH* expression expands TMM expression in embryos (Smit et al. 2023). Additionally, *MUTEpro::nucYFP* expression appeared in a similar scattering pattern but was slightly delayed compared to *SPCH* (Fig. 2, P to T). These findings align with the established notion that S, P to H regulates stomatal development one step before MUTE (MacAlister et al. 2007; Pillitteri et al. 2007).

Additionally, despite using identical promoters, the transcriptional reporter *SPCHpro::nlsGFP* displayed a markedly different expression pattern compared to the translational reporter *SPCHpro::SPCH-GFP*. As early as the heart stage, *SPCHpro::nlsGFP* was observed in the nuclei of nearly every epidermal cell (Fig. 6H). However, after the torpedo stage, the GFP signal weakened in specific regions, including the upper portion of the hypocotyl and the tip of the adaxial side of the cotyledons (Fig. 6, I to L). Notably, the abaxial side and most of the adaxial side of the cotyledons retained a strong GFP signal. Interestingly, a robust GFP signal was detected in the hypophyseal region and persisted throughout embryogenesis (Fig. 6, H to L), indicating high *SPCH* promoter activity in the root tip region.

The discrepancy between the transcriptional and translational patterns of *SPCH* during embryogenesis resembles the scenario reported in seedlings (Horst et al. 2015), suggesting the presence of post-transcriptional or post-translational regulation. Previous studies have shown that EPF signals can activate the MAPK cascade, leading to phosphorylation and subsequent degradation of transcription factors (Lampard et al. 2008; Lee et al. 2015; Qi et al. 2017). To investigate whether the EPFL8 signal specifically targets the SPCH protein, we analyzed the abundance of *SPCHpro::SPCH-GFP* in nucleus when *EPFL8* expression is induced. In transgenic plants carrying *iEPFL8* and *SPCHpro::SPCH-GFP*, we quantified GFP intensity in the nuclei of abaxial cotyledons of 4-day-postgermination (4-dpg) seedlings at various time points following estradiol or mock treatment. In the mock group, SPCH-GFP levels remained consistently high over 30 h (Fig. 6, M, N1, O1, P1, Q1, R1, and S). However, in the presence of estradiol, GFP intensity began to decrease continuously after 18 h, indicating that SPCH protein stability was reduced upon *EPFL8* induction (Fig. 6, N2, O2, P2, Q2, R2, and S). To test whether EPFL8 inhibition also occurs during embryogenesis, we first analyzed the effect of estradiol on *iEPFL8* induction in siliques. Siliques from 2 individual *iEPFL8* lines were tested, and both showed successful induction (Supplementary Fig. S3E). We then analyzed F2 embryos from F1 plants carrying both *iEPFL8* and *SPCHpro::SPCH-GFP*. Inflorescences on each F1 plant were divided into 2 groups: one treated with a mock solution and the other with estradiol. We selected the 8th silique for analysis because SPCH has low expression during early embryonic stages. Approximately one-quarter of the embryos did not show any GFP signal, likely due to genetic segregation of *SPCHpro::SPCH-GFP*. Among the embryos that did show GFP signal, one-quarter were not expected to carry *iEPFL8*. However, because these embryos could not be visually distinguished at this early developmental stage, we unbiasedly imaged all embryos with SPCH-GFP signal from the 8th siliques. Overall, the estradiol-treated group showed a significant reduction in both SPCH intensity per nucleus (Fig. 6X) and the number of GFP-positive cells per embryo (Fig. 6Y) compared to the mock group. Taken together, these results demonstrate that the EPFL8 signal reduces SPCH protein abundance in seedlings and embryos, likely by decreasing its stability.

### EPFL8 signal inhibits asymmetric division of stomatal precursor cells during embryogenesis

**Figure 7.**
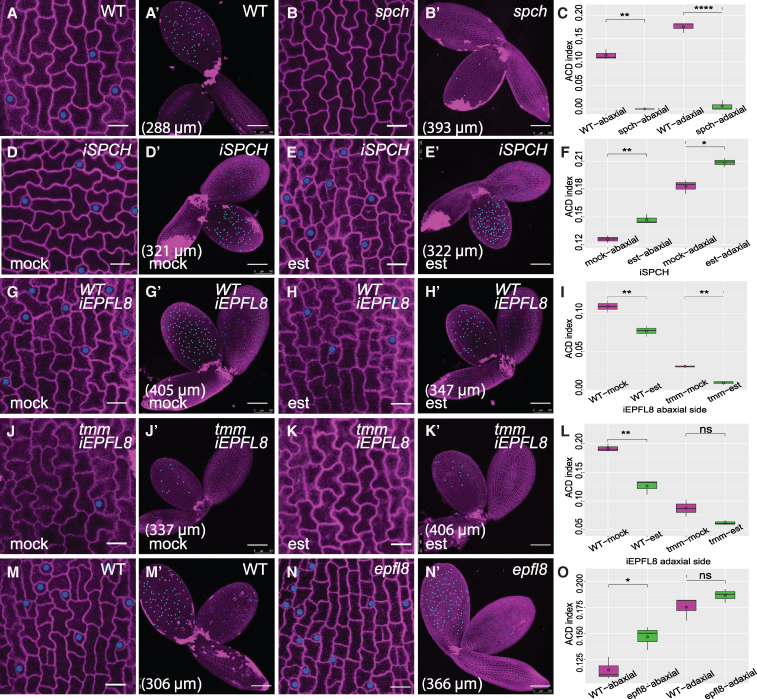
EPFL8 signal inhibits ACD events during embryogenesis. Confocal images of the abaxial side of embryo cotyledons **(A** to **N)** and corresponding entire embryo (A′ to N′) in WT seeds (A, A′), *spch* seeds (B, B′), *iSPCH* seeds whose siliques were treated with mock (D, D′) or estradiol (E, E′), *iEPFL8* seeds in WT background whose siliques were treated with mock (G, G′) or estradiol (H, H′), *iEPFL8* seeds in *tmm* background whose siliques were treated with mock (J, J′) or estradiol (K, K′), WT seeds (M, M′), and *epfl8* seeds (N, N′). PI was used to stain cell walls, ACD events were labeled in blue on the abaxial side and in cyan on the adaxial side. Scale bars: 15 µm (A to N) and 100 µm (A′ to N′). *n* = 6 for all images. (C, F, I, L, O) Boxplot quantifications of ACD index per cotyledon: Comparison between WT and *spch* mutants (C), transgenic *iSPCH* seeds treated with mock or estradiol (F), transgenic *iEPFL8* seeds in WT and *tmm* backgrounds treated with mock or estradiol (I: abaxial side, L: adaxial side), and comparison between WT seeds and *epfl8* seeds **(O)**. Statistical analysis was performed using Student's *t*-test. *P < 0.05, **P < 0.01, ****P < 0.0001, ns: not significant. *n* = 6 for all samples. For elements in all boxplots, center line: Represents the Median (Q2), which is the 50th percentile of the data. Box edges: Define the IQR, with the lower edge being the First Quartile (Q1, the 25th percentile) and the upper edge being the Third Quartile (Q3, the 75th percentile). The box contains the middle 50% of the data. Whiskers: Extend from the box to the minimum and maximum data points that are not outliers (value that is more than 1.5×IQR below Q1 or above Q3). Small internal diamond: indicates the Mean (average) value. Points (dots outside the whiskers): Outliers.

During embryogenesis, mature stomata are not formed, suggesting that additional factors regulate stomatal prepatterning beyond the mechanisms identified postgermination. Supporting this, overexpression of the transcription factors MUTE and FAMA fails to produce a detectable stomatal phenotype during embryogenesis (Smit et al. 2023), whereas SPCH promotes ACD both before and after germination (Fig. 7, A to F) (MacAlister et al. 2007; Smit et al. 2023). Our findings support the hypothesis that the SPCH protein is inhibited by the EPFL8 signal (Fig. 6). To investigate whether EPFL8 negatively regulates SPCH function, we quantified ACD events following *EPFL8* induction in WT seeds and *tmm* seeds. In WT seeds without induction, each cotyledon exhibited an average of 0.11 ACD index on the abaxial side and 0.19 on the adaxial side (Fig. 7, G, G′, I, and L). Upon estradiol-induced *EPFL8* expression, these numbers were reduced by nearly 25%, averaging 0.08 abaxially and 0.13 adaxially (Fig. 7, H, H′, I, and L). In *tmm* mutants, where EPFL8 signal is naturally enhanced, ACD index was significantly lower even without induction (Fig. 2E), averaging 0.03 abaxially and 0.08 adaxially (Fig. 7, J, J′, I, and L). When estradiol was applied to these mutants, the ACD index further decreased to 0.01 on the abaxial side and 0.05 on the adaxial side per cotyledon (Fig. 7, K, K′, I, and L), indicating reduced SPCH function. Similar results were observed in 2 additional independent iEPFL8 lines (Supplementary Fig. S4).

Additionally, we identified several *epfl8* mutants, including *epfl8-4*, a knock-out mutant (Supplementary Fig. S3F). In *epfl8-4* mutants, ACD events were significantly higher on the abaxial sides (Fig. 7, M to O). Specifically, *epfl8* cotyledon exhibited a 27% increase in abaxial ACD index compared to WT cotyledons (0.11 in WT vs. 0.15 in *epfl8*, Fig. 7, M to O). On the adaxial side, the increase in ACD index was less pronounced, with WT cotyledons averaging 0.175 ACD index versus 0.19 in *epfl8* cotyledons, a difference that was not statistically significant (Fig. 7O). The other 3 *epfl8* alleles exhibited variable increases in ACD events on both sides of the cotyledons (Supplementary Fig. S5, A and B). Collectively, these results demonstrate that EPFL8 signaling negatively regulates ACD events during embryogenesis.

### Other EPF members that contribute to stomatal precursor formation in embryogenesis

Given the potential functional redundancy among EPF family members, we examined the EPFL4/5/6 subfamily, which is expressed during embryogenesis and signal independently of TMM (Kosentka et al. 2019). As shown in Fig. 3, *EPFL5* and *EPFL6* were expressed at later embryonic stages, and RNA-seq data from Hofmann et al. (2019) (Fig. 4Z) also revealed the expression of *EPFL4 and EPFL6* during embryogenesis. To determine whether these genes influence stomatal precursor formation during embryogenesis, we quantified ACD events in the epidermal cells of mature embryos from *epfl456* triple mutants using dissected imbibed seeds. As shown in Supplementary Fig. S5, the adaxial ACD index in *epfl456* cotyledons was significantly higher than in WT (0.13 vs. 0.09, respectively; Supplementary Fig. S5, C, E, and G), whereas the increase on the abaxial side (0.08 in epfl456 vs. 0.07 in WT) was not statistically significant (Supplementary Fig. S5G). These findings suggest that, in addition to EPFL8, EPFL4/5/6 may also negatively regulate stomatal precursor formation during embryogenesis.

## Discussion

**Figure 8.**
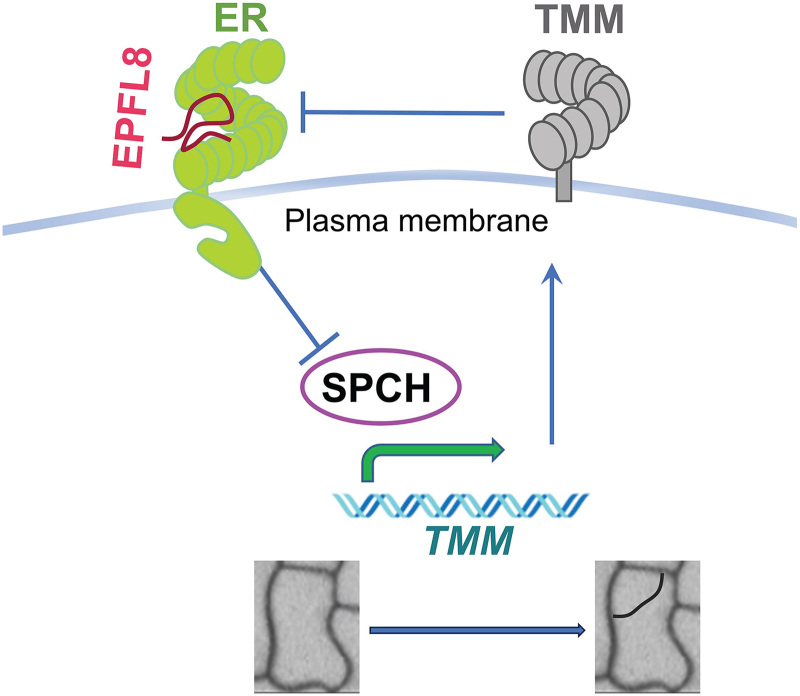
Schematic model of EPFL8 inhibition on SPCH. Early embryonic EPFL8 is perceived by the receptor kinase ER, leading to the inhibition of the SPCH protein, which is crucial for promoting the first round of asymmetric division of stomatal lineage cells during embryogenesis. The SPCH protein promotes the transcription of *TMM*, and the resulting TMM protein then impairs the EPFL8 inhibition of SPCH. The light-blue simple lines and arrows in the regulatory pathway diagram denote the flow of signal and the interaction between molecular components, where an arrow indicates a positive regulation, while a “T’ indicates negative regulation. The green curved arrow indicates that SPCH promotes the transcription of the *TMM* gene. The thick blue arrow indicates that SPCH promotes ACD during embryogenesis.

Stomata are critical for plant survival, and embryonic prepatterning of stomatal precursors primes rapid postgermination adaptation. However, the underlying mechanisms remain largely unknown. Here, we identified an EPF ligand, EPFL8, that is highly expressed during early embryogenesis. Our results indicate that EPFL8 negatively regulates stomatal prepatterning by targeting the transcription factor SPCH (Figs. 6 and 8). SPCH, in turn, promotes TMM production, a receptor protein that is dispensable for EPFL8's inhibitory signaling and may even attenuate its effect (Fig. 8). Notably, EPFL8 expression precedes that of TMM, creating a temporal conducive environment for efficient inhibition of stomatal lineage initiation (Figs. 4 and 5). Although TMM is not required for EPFL8 signal transduction, the receptor kinase ER displayed overlapping spatiotemporal expression with *EPFL8* during early embryogenesis (Figs. 4 and 5) and physically interacts with EPFL8 in the absence of TMM (Lin et al. 2017), enabling EPFL8 signaling via ER. Moreover, our modeling suggests that Lys11 on EPFL8 is critical for its reduced binding affinity to TMM (Fig. 5), allowing EPFL8 to act through ER independently of TMM to downregulate SPCH (Fig. 6). Consequently, this results in fewer ACD events and a reduced number of stomatal lineage cells during embryogenesis (Figs. 7 and 8).

Previous studies by Smit et al. (2023) showed that while the SPCH protein is first detected at the heart stage, ACD events do not occur until the torpedo stage. Moreover, even the overexpression of *SPCH* or *SCRM* fails to activate their target genes or trigger cell divisions before the late torpedo stage (Smit et al. 2023). In (Smit et al. 2023) our study, we found that although *SPCH* transcription begins broadly in nearly every protodermal cell at the heart stage, the SPCH protein is absent in most cells during the early stages (Fig. 6). Even the broad protodermal pattern of uniform SPCH protein at the cotyledon edge, as reported by Smit et al. (2023), is observed only in a subset of cells that also exhibit SPCH transcription (Fig. 6) (Smit et al. 2023). Together, these observations suggest that SPCH is inhibited either post-transcriptionally or post-translationally. The lack of ACD events during early embryogenesis may reflect the protodermal cell's inability to support SPCH or SCRM functions. Alternatively, our results indicate that the high, widespread expression of *EPFL8* in pretorpedo embryos may inhibit SPCH post-translationally, ensuring that the SPCH protein remains inactive during these early stages.

After the torpedo stage, *EPFL8* expression in cotyledons fades while SPCH accumulates in the nucleus, triggering its target genes, including *TMM* and *EPF2*, and initiating ACD events (Lau et al. 2014; Smit et al. 2023). EPF2 is recognized by a receptor complex including ER and newly synthesized TMM, which in turn modulates SPCH activity via a feedback loop that maintains SPCH homeostasis (Lampard et al. 2008; Lee et al. 2012, 2015; Horst et al. 2015). Although both EPFL8 and EPF2 inhibit SPCH during embryogenesis, their roles differ: EPFL8 is expressed prior to SPCH and broadly suppresses its initiation, whereas EPF2 is SPCH induced and relies on TMM for signaling. This feedback ensures that SPCH is not entirely inhibited but rather maintained within a localized, stable domain. In WT plants, the coordinated actions of EPFL8 and EPF2 yield appropriate stomatal precursor numbers during embryogenesis; after germination, *EPFL8* expression becomes negligible (Supplementary Fig. S3A) (Kosentka et al. 2019) while active *EPF2* fine-tunes SPCH in the stomatal lineage (Hara et al. 2009; Hunt and Gray 2009). In *tmm* mutants, however, enhanced EPFL8 inhibition during embryogenesis reduces stomatal precursor formation, and postgermination, the absence of both *EPFL8* expression and a functional EPF2 receptor complex leads to severe stomatal clustering. This disparity partially accounts for the opposing stomatal phenotypes observed in *tmm* mutants before and after germination (Figs. 1 and 2).

In *tmm* mutants, we observed a striking phenomenon where *EPFL8* expression was prolonged into a later stage in cotyledons (Fig. 4). This differential expression occurs at the late torpedo stage (Fig. 4), coinciding with the initiation of *TMM* expression by SPCH protein (Fig. 5) (Smit et al. 2023). Although TMM may play a role in some signaling pathways affecting *EPFL8* expression, *EPFL8* precedes *TMM* and is expressed in regions where *TMM* is absent, making direct regulation unlikely. Notably, *EPFL8* exhibits a strong signal at the cotyledon tips and the radicle tip (Fig. 4), which coincides with the auxin maximum point observed during embryogenesis (Cui et al. 2014). This suggests that *EPFL8* expression may be regulated by auxin or other global factors, potentially altered in the absence of *TMM*. This observation may explain the reduced ACD phenotype in *tmm* embryos and the increased ACD phenotype in *epfl8* embryos. Additionally, *EPFL4/5/6* expression emerges during later embryogenesis (Fig. 3). Although the *EPFL4/5/6* subfamily is primarily associated with inflorescence architecture and stamen elongation (Uchida et al. 2012; He et al. 2023), EPFL5 also functions to inhibit meristemoid maintenance (Niwa et al. 2013). Consistent with this role, *epfl456* triple mutants exhibit a slight increase in ACD events on the adaxial side of cotyledons (Supplementary Fig. S5), suggesting that both *EPFL8* and *EPFL4/5/6* contribute to the inhibition of ACD events in *tmm* mutants.

In the hypocotyl, *EPFL8* remains highly expressed until the mature embryo stage, at which its expression diminishes in the upper hypocotyl (Fig. 4), coinciding with the appearance of *SPCH* and *TMM* in the same region (Figs. 5 and 6). After germination, *EPFL8* expression vanishes (Supplementary Fig. S3A) (Kosentka et al. 2019) while *EPFL4/5/6* remain highly expressed, particularly in hypocotyl (Abrash and Bergmann 2010; Abrash et al. 2011). In *tmm* mutants, sustained high expression of *EPFL8* and *EPFL4/5/6* in the hypocotyl results in a near-complete absence of stomatal phenotype both before and after germination (Yang and Sack 1995). Notably, *SPCH* is strongly expressed in the radicles throughout embryogenesis, but neither SPCH protein nor its target TMM proteins are detected there, suggesting that SPCH may play a role in radicle beyond stomatal development. *EPFL8* also showed strong expression in the radicles throughout embryogenesis. To our knowledge, *EPFL8* is the only *EPFL* family member with such high expression in radicles during embryogenesis. After germination, *EPFL9/STOMAGEN* is reported to be transcribed in roots, where it participates in redox-mediated cortex regulation (Cui et al. 2014). Smit and colleagues suggest that stomatal-related genes may also be linked to cell proliferation (Smit et al. 2023). Whether EPFL8 and SPCH genetically interact in radicles and whether they are involved in radicle development remains unclear.

An intriguing phenomenon we observed was the initial expression of stomatal-related genes, including *MUTE*, *TMM*, and *ERL1*, which first appeared in the tip region of the embryo cotyledons (Figs. 2 and 5). While *SPCH* is ubiquitously expressed, its protein initially accumulates in the same cotyledon tip region (Fig. 6). Based on the location, these cells are likely precursor cells to hydathode pores (Cerutti et al. 2017; Jauneau et al. 2020). Hydathode pores are evolutionarily related stomata-like structures in the epithemal hydathode that always open to release water and dissolved solutes from xylems, a process called guttation (Cerutti et al. 2019). Previous studies have reported that several markers for stomatal identity or differentiation are shared with the precursor cells of hydathode pores (Pillitteri et al. 2008; Cerutti et al. 2017). Our results also suggest that hydathode pores and stomata share a common origin in *Arabidopsis* embryogenesis, with hydathode pore development initiating before stomatal development. In *Arabidopsis*, hydathode pores typically cluster together to facilitate continuous guttation (The PyMOL Molecular Graphics System, Version 3.0 Schrödinger, LLC; Pillitteri and Dong 2013), whereas stomata follow the one-cell-spacing rule to enable efficient gas exchange, adapting to specific environmental conditions. Interestingly, although the stomatal index is significantly altered in *tmm* mutants (Figs. 2 and 7), the initiation of stomatal marker proteins at the hydathode pore positions is minimally altered (Fig. 2). This suggests that EPFL8 signaling may play distinct roles in hydathode pore and stomatal development. Specifically, EPFL8 appears to inhibit stomatal development more strongly than hydathode pore development. Alternatively, hydathode pore development may possess protective mechanisms that mitigate the inhibitory effects of EPF signals, including those enforcing the one-cell-spacing rule.

Finally, we observed a striking difference in stomatal precursor density between the 2 sides of the cotyledons during embryogenesis. Stomatal precursor cells emerge earlier on the adaxial side of the cotyledon, where their density is nearly twice that of the abaxial side in mature embryos (Figs. 2 and 7). This asymmetry persists after germination, as stomatal distribution remains distinct on both sides of the leaf in *A. thaliana*. In the plant kingdom, stomatal distribution patterns vary across species. Amphistomatous plants, like *A. thaliana*, have stomata on both leaf surfaces, while hypostomatous plants only have stomata on the lower surface, and epistomatous plants restrict stomata to the upper surface. These patterns suggest that leaf polarity plays an important role in stomatal development. Despite this clear connection, the key regulators linking leaf polarity to stomatal development remain largely unknown. Investigating stomatal development in plant materials with manipulated leaf polarity or in species with naturally diverse leaf polarity presents an exciting avenue for future research.

## Materials and methods

### Key resources table

See Supplementary Table S1.

## Resource availability

### Lead contact

Further information and requests for resources and reagents should be directed to and will be fulfilled by the lead contact, Xingyun Qi (Xingyun.qi@rutgers.edu).

### Materials availability

All unique/stable reagents generated in this study are available from the Lead Contact without restriction.

### Plant materials and growth conditions

The *A. thaliana* Columbia (Col) accession was used as the WT control. The following reporter lines were utilized as previously reported: *tmm-KO* (Hara et al. 2007), spch (Pillitteri et al. 2007), *EPF1pro:erGFP*, *MUTEpro:MUTE-GFP*, *EPFL4pro:EGFP-GUS*, *EPFL5pro:EGFP-GUS*, *EPFL6pro:EGFP-GUS* (Kosentka et al. 2019), *ERL1pro:ERL1-YFP* (Qi et al. 2017), *ERpro:ER-YFP*, *TMMpro:TMM-YFP*, *SPCHpro:SPCH-GFP* (Pillitteri et al. 2007), *SPCHpro:nlsGFP* (Horst et al. 2015), and *Est:SPCH* (*iSPCH*) (Lee et al. 2012). The following mutants used in the study were obtained from Arabidopsis Biological Resource Center (Ohio State University): *epfl8-1 (sail_215_A07)*, *epfl8-4 (salk_015571)*, *epfl8-5 (salk_040475)*, *epfl8-6 (salk_013193)*, *epfl4/cll2-1 (salk_071065)*, *epfl5/cll1-1 (salk_005080)*, and *epfl6/chal-2 (salk_072522)*. Plants were grown at 22 °C under long-day conditions (16 h light/8 h dark).

### Plasmid construction and generation of transgenic plants

The following plasmids were generated for this study*: iEPFL8*, *EPFL8pro:GFP*, and *EPFL8pro:GUS*. To construct *EPFL8pro:GFP* and *EPFL8pro:GUS*, the *EPFL8* promoter region, spanning −2001 bp to −1 bp upstream of the start codon, was PCR amplified with *Xho*I and *Spe*I restriction sites at the 5′ and 3′ ends, respectively. The amplified PCR fragment was subcloned into pKUT612. Subsequently, an LR reaction was performed between the resulting plasmid and the destination vector pGWB3 (for *EPFL8pro:GUS*) or pGWB4 (for *EPFL8pro:GFP*), producing constructs in which the *EPFL8* promoter was fused to GUS or GFP at the start codon. For the *iEPFL8* construct, the open reading frame of *EPFL8,* including the stop codon, was cloned with *Xho*I and *Spe*I restriction sites at the 5′ and 3′ ends. The fragment was then directly assembled into the destination vector pER8 via recombination (Zuo et al. 2000). The *35Spro:EPFL4-3xFLAG*, *35Spro:EPFL5-3xFLAG*, and *35Spro:EPFL6-3xFLAG* were generated by the TORII lab using LR reaction between the pENTR-D-TOPO carrying the corresponding EPFL cDNA and pGWB2. For additional details about the plasmids used, refer to Supplementary Table S2.

Plasmids were transformed into *Agrobacterium tumefaciens* strain GV3101/pMP90 and subsequently introduced into *A. thaliana* via the floral dipping method. The following transgenic lines were generated in this study: *iEPFL8*, *EPFL8pro:GFP*, *EPFL8pro:GUS*, *35Spro:EPFL4-3xFLAG*, *35Spro:EPFL5-3xFLAG*, and *35Spro:EPFL6-3xFLAG.* Over 10 independent lines for each plasmid were characterized for phenotypes and reporter gene expressions. Established transgenic lines were used for genetic crosses with mutants or other reporter lines, including *SPCHpro:SPCH-GFP x epfl8-4* and *SPCHpro::SPCH-GFP x iEPFL8.* For primer DNA sequence used for plasmid construction, see Supplementary Table S3.

### Estradiol application

Estradiol induction was performed as described previously, with modifications (Lee et al. 2012). The induction of *iEPFL8* was validated through seedling bioassays (Lee et al. 2015). Fifty seedlings were immersed in 1/2 MS solutions supplemented with either DMSO (mock) or 10 µM estradiol (Est) and incubated under the described growth conditions with shaking. For RT-qPCR analysis, 3 replicates of 50 *iEPFL8* seedlings per treatment (mock or estradiol) were collected 8 h post-treatment for total RNA isolation. For confocal imaging, homozygous transgenic *iEPFL8* lines were kept on 1/2 MS agar plates for 2 d stratification at 4 °C, followed by 1 d at 22 °C. Subsequently, 20 seedlings per genotype per treatment were immersed in 1/2 MS liquid media containing 10 µM estradiol (Sigma-Aldrich, E2758) or DMSO (mock) for 3 d. For embryo-stage treatment, flowers were dipped into 1/2 MS solutions supplemented with DMSO (mock) or 10 µM estradiol (Est) every other day until seeds mature. Embryos were then dissected from siliques, and stomatal phenotypes were analyzed using a confocal microscope. For SPCH-GFP quantification in embryos, we grew F1 plants of *iEPFL8* *×* *SPCH-GFP* until inflorescences developed. Branches from the same F1 plant were split into 2 groups: one treated with DMSO solution and the other with 10 µM estradiol. Because *SPCH* expression is low at early stages, we selected the 8th silique for embryo dissection. Embryos were collected after 24 h of treatment and z-stack imaged using confocal microscopy. In total, 3 F1 plants were analyzed.

### Embryo dissection

We followed the method described by Raissig et al. (2013) with modifications: Remove seeds from the corresponding siliques (5th to 8th) using an inverted microscope equipped with 10× and 20× magnification objectives. Use a needle to carefully break and remove the seed coat. Transfer the embryos into a 5% glycerol on the slide and gently cover the samples with a cover slip. For younger siliques (3rd to 5th), dissect seeds from siliques and directly transfer them into 5% glycerol on the slide, cover the samples with a cover slip, and release the embryos by gently pressing the cover slip. The force required may vary and should be determined by each user through trial and adjustment. In general, the globular, heart, torpedo, upright, and walk-stick stages were dissected from the 4th to the 8th silique, respectively. Embryo morphology and cotyledon length were further analyzed to accurately identify each stage. For RT-qPCR analysis, seeds were collected from 3rd to 6th siliques separately. For each silique stage, seeds were harvested from approximately 100 plants. All the seeds collected were kept in liquid nitrogen throughout the harvesting process to maintain RNA integrity.

### Phylogenetic analysis

The 11 EPF/EPFL sequences were retrieved from the genomic database for *A. thaliana* (The Arabidopsis Information Resource, TAIR). Amino acid sequences corresponding to the full-length peptide regions of these 11 ligands were aligned using the ClustalW program (Thompson et al. 1994). Phylogenetic reconstructions were conducted using the “build” function of ETE3 3.1.2 (Huerta-Cepas et al. 2016), implemented on the GenomeNet platform (https://www.genome.jp/tools/ete/). A maximum likelihood tree was inferred using RAxML v8.2.11 with the PROTGAMMAJTT model and default parameters (Stamatakis 2014). Branch supports were computed based on 100 bootstrapped trees.

### Homology modeling

Structural models of EPFL8 in complex with ERL1 and ERL1-TMM were generated using MODELLER version 9.16 (Sali and Blundell 1993), with the crystal structure of EPF1 in complex with ERL1-TMM (PDB code 5xjo) (Nagel et al. 2017) serving as the template. The EPFL8 peptide was aligned to the EPF1 template, as shown in Fig. 5, A, B, and B′. Model optimization was performed using MODELLER's AutoModel class with default parameters. Electrostatic potential surfaces were computed using the APBS and PDB2PQR software (Jurrus et al. 2018). Molecular models were rendered with PyMol (The PyMOL Molecular Graphics System, Version 3.0 Schrödinger, LLC).

### RT-qPCR

For *iEPFL8* induction analysis, homozygous transgenic lines for iEPFL8 were grown on 1/2 MS agar media for 4 d. For each replicate, 40 to 50 seedlings were immersed in 1/2 MS liquid media containing either 10 μM estradiol or DMSO (mock) for 8 h. For *EPFL8* expression analysis, seeds were collected from 3rd to 6th siliques separately. For each silique stage, seeds were harvested from approximately 100 plants. All the seeds collected were kept in liquid nitrogen throughout the harvesting process to maintain RNA integrity. Total RNA was extracted using the RNeasy Plant Mini Kit (Qiagen, 74904) with on-column DNase I digestion (Qiagen, 79254) performed according to the manufacturer's instructions. After normalization, 1 μg of RNA was reverse-transcribed to cDNA using the iScript cDNA synthesis kit (Bio-Rad, 1708891), following the manufacturer's protocol. The resulting first-strand cDNA was diluted 1:7 in double-distilled water and used as the template for real-time qPCR. RT-qPCR was performed as described in the chromatin immunoprecipitation section. To generate a standard curve and calculate the gene expression quantities, at least four 10-fold serial dilutions were amplified using the same primers. Relative expression levels were determined by normalizing the expression of target genes to *ACT2*. Three biological replicates were conducted. For oligo DNA primer sequences used in RT-qPCR, see Supplementary Table S3.

### Confocal microscopy and time-lapse imaging

The Leica SP8 inverted confocal microscope (Solms, Germany) was used for imaging. Time-lapse imaging of cotyledons expressing *EPF1pro:erGFP* was conducted as previously described, using a 20x/0.8 Apochromat lens (x1 zoom) on the Leica SP8 (Pillitteri et al. 2011; Peterson and Torii 2012; Qi et al. 2017). For imaging PI-stained samples and transgenic plants with GFP or YFP signals, cotyledons or dissected embryos were observed with the same 20x/0.8 Apochromat lens and a Hy-D detector on the Leica SP8. PI was excited with a 555 nm laser, GFP with a 488 nm laser, and YFP with a 514 nm laser. Emission filters were set to 600 to 675 nm for PI staining, 500 to 530 nm for GFP, and 550 to 600 nm for YFP. Z-stack projection images were acquired at 0.99 µm intervals, capturing the entire thickness of the cell. For qualitative image presentation, Adobe Photoshop CS6 was used to adjust contrast and brightness uniformly.

### Quantitative analysis and statistics

Leica LAS AF software and Imaris ver. 8.1.3 (Bitplane) were used for postacquisition image processing. Quantitative analysis of SPCH signal intensity was performed with Imaris ver. 8.1.3 as follows. A comprehensive series of Z-stack confocal images (∼17 layers) encompassing the entire meristemoids was used for surface rendering in the red or green channel to capture *SPCH-GFP* expressing nuclei in 3D space for each time point in the time course. A sphericity cut-off value of 0.85 was applied to effectively exclude objects with nonspecific signals. For each nucleus, the intensity sum and standard deviation were calculated. Statistical analyses were carried out using R ver. 3.3.1, and data visualization was achieved using the ggplot2 package in R or Microsoft Excel.

### Accession numbers

Sequence data from this article can be found in the TAIR data libraries under the following accession numbers: TMM (AT1G80080), ER (AT2G26330), ERL1 (AT5G62230), ERL2 (AT5G07180), EPF1 (AT2G20875), EPFL4 (AT4G14723), EPFL5 (AT3G22820), EPFL8 (AT1G80133), EPFL6 (AT2G30370), SPCH (AT5G53210), and MUTE (AT3G06120).

## Supplementary Material

kiaf487_Supplementary_Data

## Data Availability

All data are incorporated into the article and its online supplementary material.

## Supplementary data

The following materials are available in the online version of this article.

**Supplementary Figure S1.** EPFL4/5/6 inhibits stomatal formation independent of TMM.

**Supplementary Figure S2.** EPFL8 falls in the subfamily of EPFL4/5/6.

**Supplementary Figure S3.** EPFL8 expression in various plant materials.

**Supplementary Figure S4.** EPFL8 signal inhibits ACD events during embryogenesis.

**Supplementary Figure S5.** EPFL8 and EPFL4/5/6 signal inhibits ACD events during embryogenesis.

**Supplementary Table S1.** Key resources table.

**Supplementary Table S2.** Plasmids used in this study.

**Supplementary Table S3.** Primers used in this study.
