# Supplementary material for: The peptide EPFL8 represses embryonic stomatal precursor formation independently of TOO MANY MOUTHS in Arabidopsis
Source: Plant Physiol. 2025 Oct 6;199(2):kiaf487. doi: 10.1093/plphys/kiaf487 (PMC12620614; doi:10.1093/plphys/kiaf487)
Supplement: kiaf487_Supplementary_Data [file kiaf487_Supplementary_Data.pdf]

Supplementary information

Figures S1-S5 and Tables S1-S3

Supplementary Figure S1. EPFL4/5/6 inhibit stomatal formation independent of TMM. Stomatal phenotype of 4 days-post-germination plants overexpressing *EPFL4, EPFL5,* and *EPFL6* in wild-type background (A, C, E, G) or the *tmm* mutant background (B, D, F, H). Propidium iodide (PI) was used to stain cell walls, Mature stomata were highlighted in green and meristemoids in red. oe: overexpression. Scale Bars: 25 µm for all panels. N = 6 for all images.

Supplementary Figure S2. EPFL8 falls in the subfamily of EPFL4/5/6. (A) The phylogenetic tree of the 11 EPF members in *Arabidopsis thaliana*. The group that requires TMM for signal transduction is highlighted in magenta. The group that does not require TMM for signal transduction is highlighted in cyan. Bootstrap support values (in brackets) and branch lengths (in parentheses or preceding brackets) are shown next to each branch. The scale at the top of the tree (e.g., 0, 0.2, 0.4, ..., 2.6) indicates the expected number of amino acid substitutions per site. (B) The model of EPFL8 structure generated by the PDB 3D Protein Feature View. The magenta arrow points to the location of Lys11 and Arg13 on EPFL8. (C) The alignment of the mature peptide sequences of the 11 EPF members in *Arabidopsis thaliana*. Red and blue indicate highly and partially conserved amino acids, respectively. Lys11 and Arg13 are highlighted in magenta.

Supplementary Figure S3. The *EPFL8* expression in various plant materials. (A) An image of a seedling expressing *EPFL8pro::GUS.* The GUS signal driven by the *EPFL8* promoter is shown in blue, if any. Scale bars:5 mm. N = 6. (B) The RT-qPCR result showing the relative expression level of *EPFL8* in seeds from different siliques of the wild-type plants. N = 3; Normalization: actin; Error bars: standard error. (C) The RT-qPCR result showing the relative expression level of *EPFL8* in wild-type or *tmm* mutants using seeds from silique #3, #4 and silique #5, #6. Fold change in comparison to the wild-type silique #3, #4 sample is shown. N = 3; Normalization: actin; Error bars: standard error. (D) The RT-qPCR result showing the relative expression level of *EPFL8* in 4 dpg transgenic plants bearing estradiol-inducible EPFL8 (*iEPFL8)* in wild-type or *tmm* background treated with mock or 10 µM estradiol (Est) for 8 hours. Fold change in comparison to the mock sample is shown. N = 3; Normalization: actin; Error bars: standard error. (E) The RT-qPCR result showing the relative expression level of EPFL8 in siliques from two individual iEPFL8-positive transgenic lines treated with mock or 10 µM estradiol (Est) for 5 hours. Fold change in comparison to the mock sample is shown. N = 3; Normalization: actin; Error bars: standard error. (F) The RT-qPCR result showing the relative expression level of EPFL8 in different T-DNA insertion mutants. Fold change

in comparison to the wild-type sample is shown. N = 3; Normalization: actin; Error bars: standard error.

Supplementary Figure S4. The EPFL8 signal inhibits ACD events during embryogenesis. Boxplot quantifications of asymmetric cell division (ACD) index on the abaxial side (A, C) and adaxial side (B, D) of cotyledons of two independent transgenic lines of iEPFL8: #1 (A, B) and #2 (C, D). Satistical analysis was performed using Student's t-test. * : $p<0.05$, ** : $p<0.01$, ns : not significant. n = 6 for all samples. Center Line: Represents the Median (Q2), which is the 50th percentile of the data. Box Edges: Define the Interquartile Range (IQR), with the lower edge being the First Quartile (Q1, the 25th percentile) and the upper edge being the Third Quartile (Q3, the 75th percentile). The box contains the middle 50% of the data. Whiskers: Extend from the box to the minimum and maximum data points that are not outliers (value that is more than 1.5×IQR below Q1 or above Q3). Small Internal Diamond: indicates the Mean (average) value.

Supplementary Figure S5. The EPFL8 and EPFL4/5/6 signal inhibits ACD events during embryogenesis. (A, B) Boxplot quantifications of asymmetric cell division (ACD) index on the abaxial side (A) and adaxial side (B) of cotyledons of wild-type plants and three epfl8 mutants. Statistical analysis was performed using Student's t-test. * : $p<0.05$, ns : not significant. n = 3 for all samples. (C-F) Confocal images of the entire embryo (C, E) and corresponding abaxial side of embryo cotyledons (D, F) in wild-type (C, D), and *epfl456* triple mutant (E, F). Propidium iodide (PI) was used to stain cell walls, ACD events were labelled in blue on the abaxial side and in cyan on the adaxial side. Scale bars: 15 μm (D, F) and 100 μm (C, E). n = 6 for all images. (G) Boxplot quantifications of ACD index on two sides of cotyledons of wild-type plants and *epfl456* mutants. Statistical analysis was performed using Student's t-test. **** : $p<0.0001$, ns : not significant. n = 6 for all samples. Center Line: Represents the Median (Q2), which is the 50th percentile of the data. Box Edges: Define the Interquartile Range (IQR), with the lower edge being the First Quartile (Q1, the 25th percentile) and the upper edge being the Third Quartile (Q3, the 75th percentile). The box contains the middle 50% of the data. Whiskers: Extend from the box to the minimum and maximum data points that are not outliers (value that is more than 1.5×IQR below Q1 or above Q3). Small Internal Diamond: indicates the Mean (average) value. Points (Dots Outside the Whiskers): Outliers.

Supplementary Figure S1

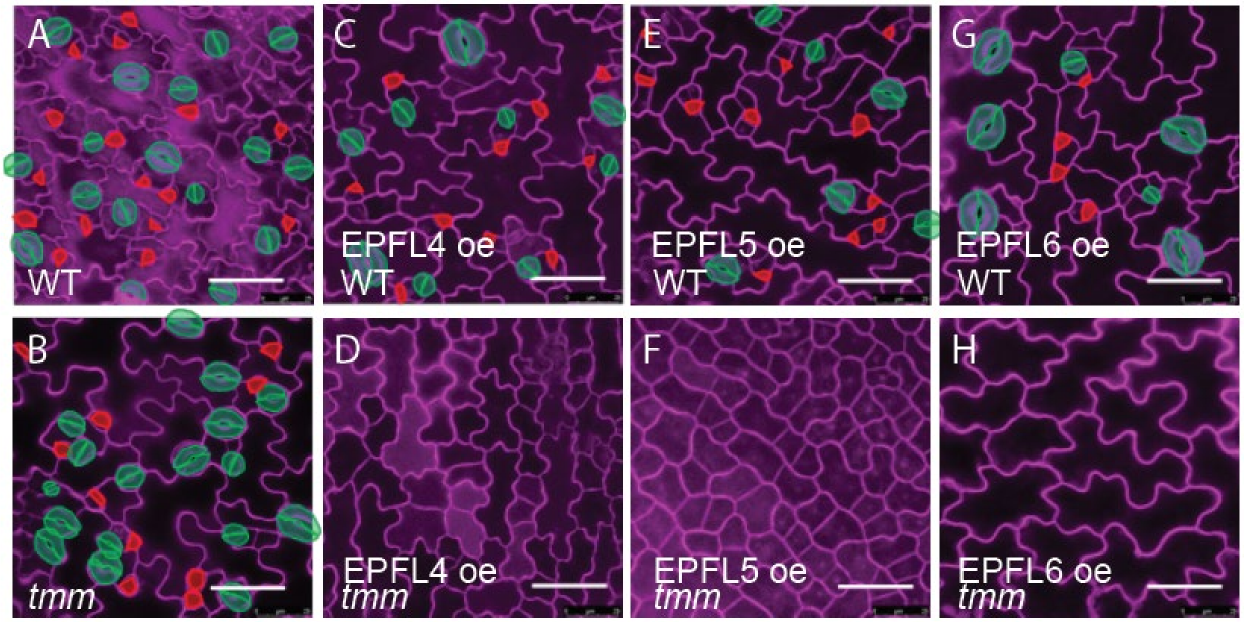

Supplementary Figure S1. EPFL4/5/6 inhibit stomatal formation independent of TMM. Stomatal phenotype of 4 days-post-germination plants overexpressing *EPFL4, EPFL5,* and *EPFL6* in wild-type background (A, C, E, G) or the *tmm* mutant background (B, D, F, H). Propidium iodide (PI) was used to stain cell walls, Mature stomata were highlighted in green and meristemoids in red. oe: overexpression. Scale Bars: 25 μm for all panels. N = 6 for all images.

Supplementary Figure S2

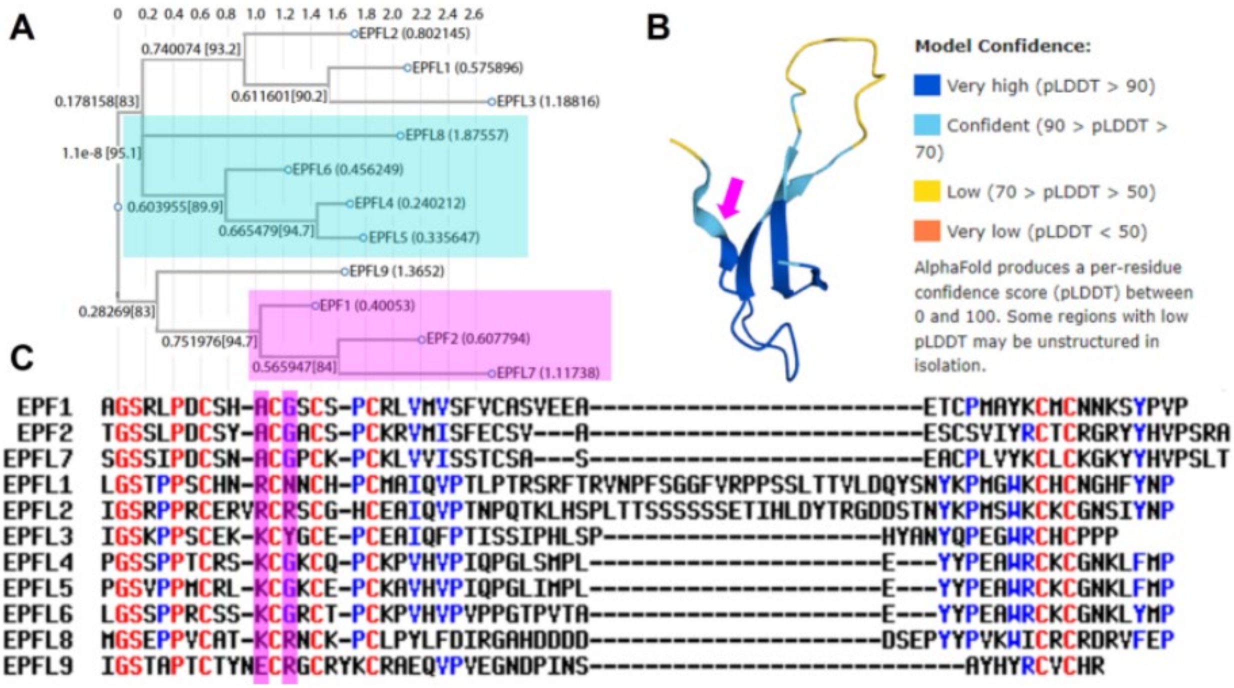

Supplementary Figure S2. EPFL8 falls in the subfamily of EPFL4/5/6. (A) The phylogenetic tree of the 11 EPF members in *Arabidopsis thaliana*. The group that requires TMM for signal transduction is highlighted in magenta. The group that does not require TMM for signal transduction is highlighted in cyan. Bootstrap support values (in brackets) and branch lengths (in parentheses or preceding brackets) are shown next to each branch. The scale at the top of the tree (e.g., 0, 0.2, 0.4, ..., 2.6) indicates the expected number of amino acid substitutions per site. (B) The model of EPFL8 structure generated by the PDB 3D Protein Feature View. The magenta arrow points to the location of Lys11 and Arg13 on EPFL8. (C) The alignment of the mature peptide sequences of the 11 EPF members in *Arabidopsis thaliana*. Red and blue indicate highly and partially conserved amino acids, respectively. Lys11 and Arg13 are highlighted in magenta.

Supplementary Fig 3

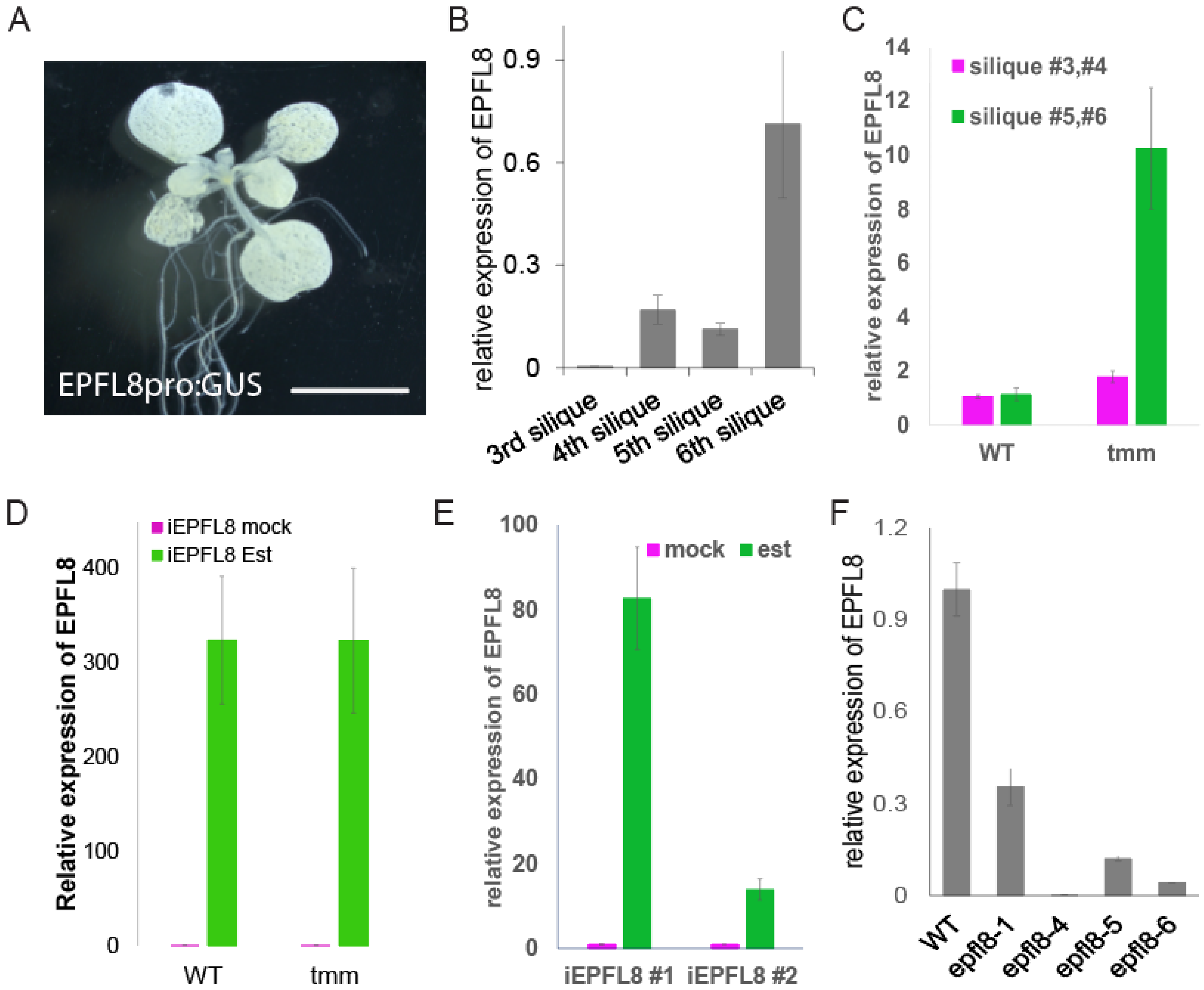

Supplementary Figure S3. The *EPFL8* expression in various plant materials. (A) An image of a seedling expressing *EPFL8pro::GUS.* The GUS signal driven by the *EPFL8* promoter is shown in blue, if any. Scale bars:5 mm. N = 6. (B) The RT-qPCR result showing the relative expression level of *EPFL8* in seeds from different siliques of the wild-type plants. N = 3; Normalization: actin; Error bars: standard error. (C) The RT-qPCR result showing the relative expression level of *EPFL8* in wild-type or *tmm* mutants using seeds from silique #3, #4 and silique #5, #6. Fold change in comparison to the wild-type silique #3, #4 sample is shown. N = 3; Normalization: actin; Error bars: standard error. (D) The RT-qPCR result showing the relative expression level of *EPFL8* in 4 dpg transgenic plants bearing estradiol-inducible EPFL8 (*iEPFL8)* in wild-type or *tmm* background treated with mock or 10 µM estradiol (Est) for 8 hours. Fold change in comparison to the mock sample is shown. N = 3; Normalization: actin; Error bars: standard error. (E) The RT-qPCR result showing the relative expression level of EPFL8 in siliques from two individual iEPFL8-positive transgenic lines treated with mock or 10 µM estradiol (Est) for 5 hours. Fold change in comparison to the mock sample is shown. N = 3; Normalization: actin; Error bars: standard error. (F) The RT-qPCR result showing the relative expression level of EPFL8 in different T-DNA insertion mutants. Fold change in comparison to the wild-type sample is shown. N = 3; Normalization: actin; Error bars: standard error.

Supplementary Figure S4

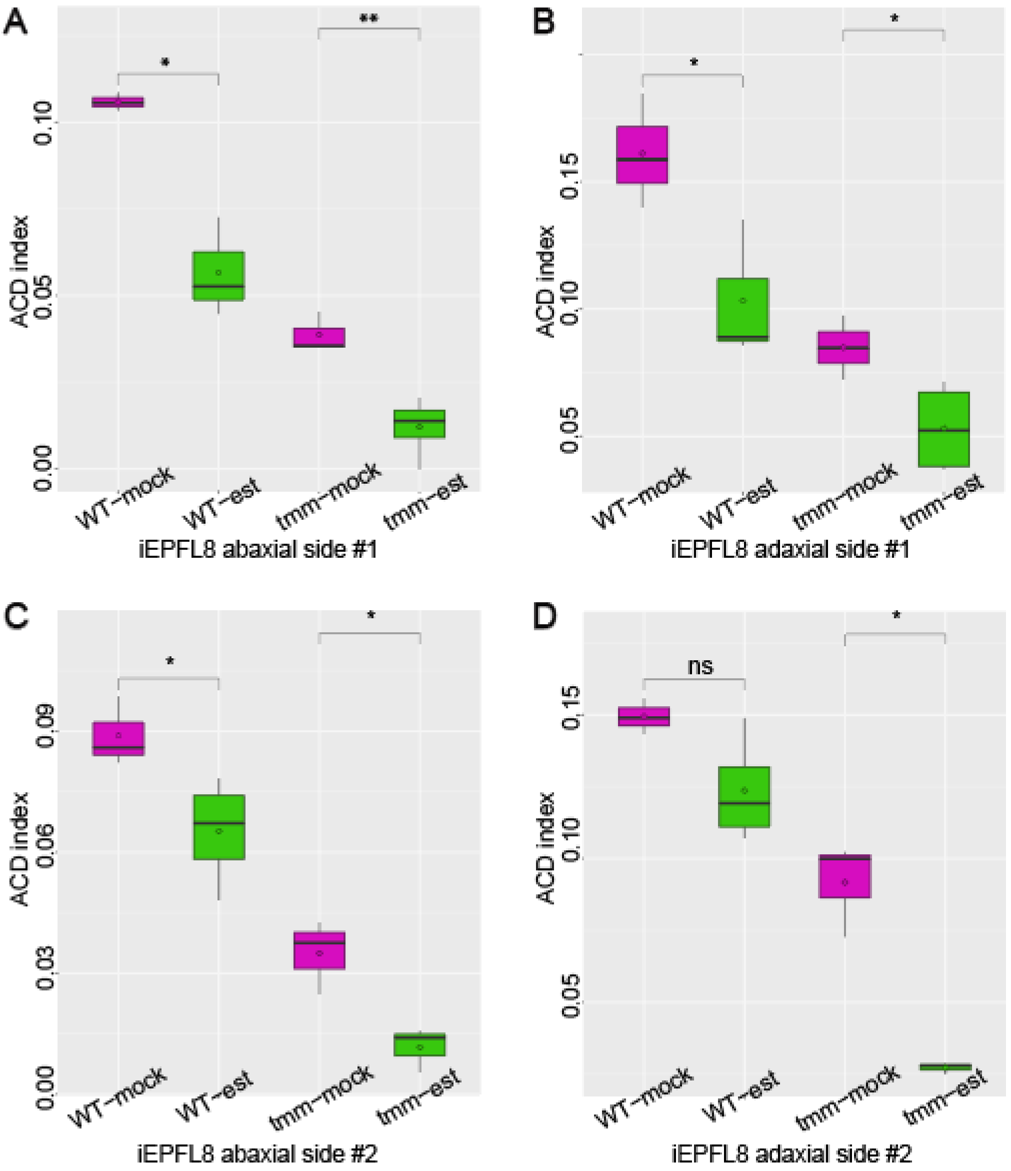

Supplementary Figure S4. The EPFL8 signal inhibits ACD events during embryogenesis. Boxplot quantifications of asymmetric cell division (ACD) index on the abaxial side (A, C) and adaxial side (B, D) of cotyledons of two independent transgenic lines of *iEPFL8*: #1 (A, B) and #2 (C, D). Satistical analysis was performed using Student's t-test. * : $p<0.05$, ** : $p<0.01$, ns : not significant. n = 6 for all samples. Center Line: Represents the Median (Q2), which is the 50th percentile of the data. Box Edges: Define the Interquartile Range (IQR), with the lower edge being the First Quartile (Q1, the 25th percentile) and the upper edge being the Third Quartile (Q3, the 75th percentile). The box contains the middle 50% of the data. Whiskers: Extend from the box to the minimum and maximum data points that are not outliers (value that is more than 1.5×IQR below Q1 or above Q3). Small Internal Diamond: indicates the Mean (average) value.

Supplementary Figure S5

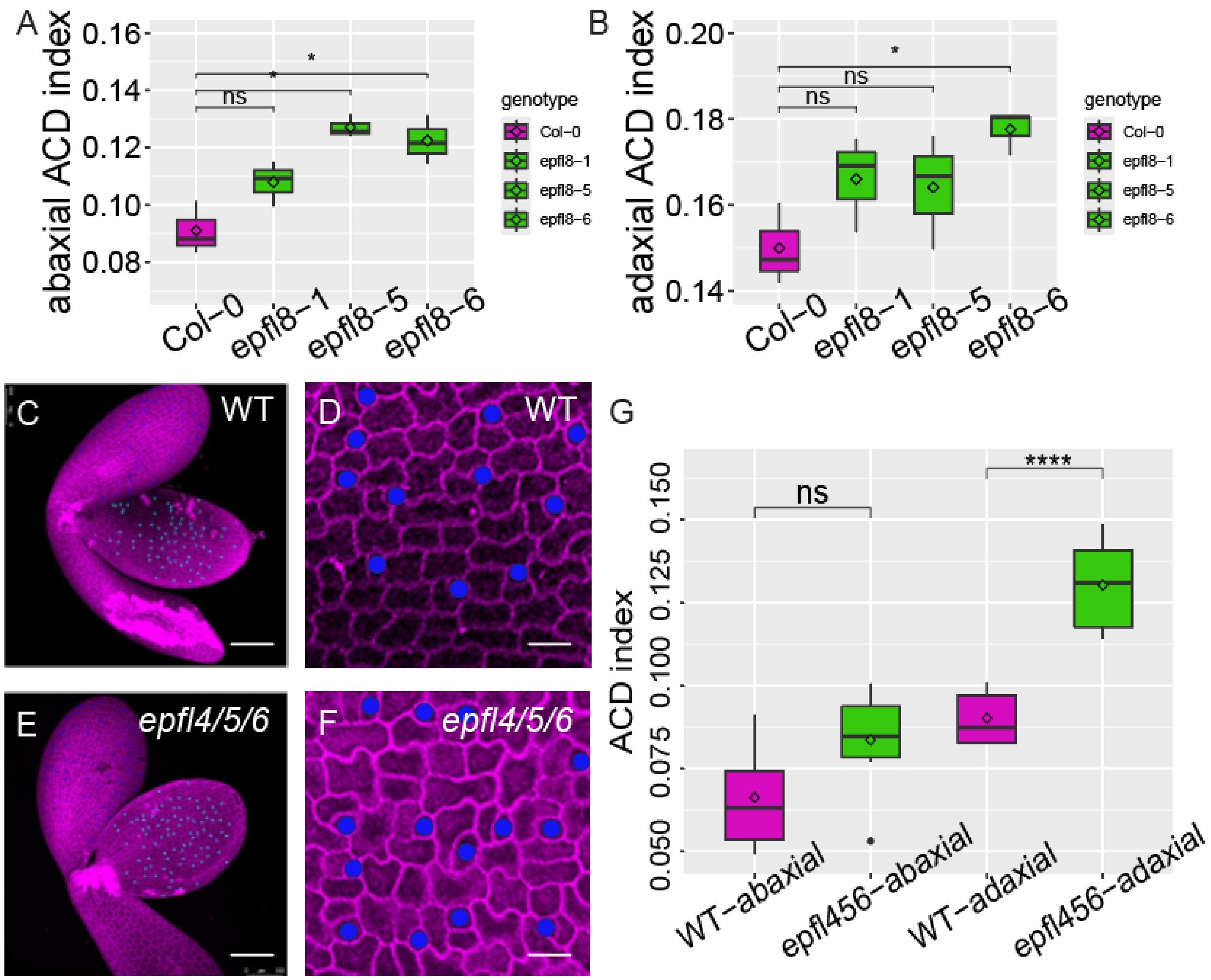

Supplementary Figure S5. The EPFL8 and EPFL4/5/6 signal inhibits ACD events during embryogenesis. (A, B) Boxplot quantifications of asymmetric cell division (ACD) index on the abaxial side (A) and adaxial side (B) of cotyledons of wild-type plants and three epfl8 mutants. Statistical analysis was performed using Student's t-test. * : p<0.05, ns : not significant. n = 3 for all samples. (C-F) Confocal images of the entire embryo (C, E) and corresponding abaxial side of embryo cotyledons (D, F) in wild-type (C, D), and *epfl456* triple mutant (E, F). Propidium iodide (PI) was used to stain cell walls, ACD events were labelled in blue on the abaxial side and in cyan on the adaxial side. Scale bars: 15 μm (D, F) and 100 μm (C, E). n = 6 for all images. (G) Boxplot quantifications of ACD index on two sides of cotyledons of wild-type plants and *epfl456* mutants. Statistical analysis was performed using Student's t-test. **** : p<0.0001, ns : not significant. n = 6 for all samples. Center Line: Represents the Median (Q2), which is the 50th percentile of the data. Box Edges: Define the Interquartile Range (IQR), with the lower edge being the First Quartile (Q1, the 25th percentile) and the upper edge being the Third Quartile (Q3, the 75th percentile). The box contains the middle 50% of the data. Whiskers: Extend from the box to the minimum and maximum data points that are not outliers (value that is more than 1.5×IQR below Q1 or above Q3). Small Internal Diamond: indicates the Mean (average) value. Points (Dots Outside the Whiskers): Outliers.

Supplementary Table S1 **Key resources table**

**Plant material name**	**resourse**
tmm-KO	Hara et al., 2007, Genes & development
spch	Pillitteri et al., 2007, Nature
EPF1pro::erGFP	Qi et al., 2017, Elife
MUTEpro::MUTE-GFP	Qi et al., 2017, Elife
EPFL4pro::EGFP-GUS	Kosentka et al., 2019, Plant Physiol
EPFL5pro::EGFP-GUS	Kosentka et al., 2019, Plant Physiol
EPFL6pro::EGFP-GUS	Kosentka et al., 2019, Plant Physiol
ERL1pro::ERL1-YFP	Qi et al., 2017, Elife
ERpro::ER-YFP	Pillitteri et al., 2007, Nature
TMMpro::TMM-YFP	Pillitteri et al., 2007, Nature
SPCHpro::SPCH-GFP	Pillitteri et al., 2007, Nature
SPCHpro::nucYFP	Horst et al., 2015, PloS Genet
Est::SPCH (iSPCH)	Lee et al., 2012, Genes & development
epfl8-1 (sail_215-A07)	Arabidopsis Biological Resource Center (Ohio State University)
epfl8-4 (salk_015571)	Arabidopsis Biological Resource Center (Ohio State University)
epfl8-5 (salk_040475)	Arabidopsis Biological Resource Center (Ohio State University)
epfl8-6 (salk_013193)	Arabidopsis Biological Resource Center (Ohio State University)
epfl4/cll2-1 (salk_071065)	Arabidopsis Biological Resource Center (Ohio State University)
epfl5/cll1-1 (salk_005080)	Arabidopsis Biological Resource Center (Ohio State University)
epfl6/chal-2 (salk_072522)	Arabidopsis Biological Resource Center (Ohio State University)
iEPFL8	generated in reseachers' lab of this study

EPFL8pro:GFP	generated in reseachers' lab of this study
EPFL8pro:GUS	generated in reseachers' lab of this study
35Spro::EPFL4-3XFLAG	generated in reseachers' lab of this study
35Spro::EPFL5-3XFLAG	generated in reseachers' lab of this study
35Spro::EPFL6-3XFLAG	generated in reseachers' lab of this study
SPCHpro::SPCH-GFP x epfl8-4	generated in reseachers' lab of this study
SPCHpro::SPCH-GFP x iEPFL8	generated in reseachers' lab of this study

Supplementary Table S2 Plasmids used in this study

Plasmid ID	description	Insertion	Vector	Process
**iEPFL8**	inducible EPFL8 by applying estradiol	EPFL8 cds	pER8	PCR, ligation
**EPFL8pro::GFP**	2kb EPFL8 promoter driven GFP	2kb EPFL8 promoter	pGWB4	PCR, ligation,Gateway recombination
**EPFL8pro::GUS**	2kb EPFL8 promoter driven GUS	2kb EPFL8 promoter	pGWB3	PCR, ligation,Gateway recombination

Supplementary Table S3 Primers used in this study

Gene names	Primer names	Sequences (5' to 3')	Purpose	note
EPFL8	EPFL8-common-LP	CACGTTGACCATAACCACATG	mutant genotyping	
	EPFL8-common-RP	GTGGTGCTCATGATGATGATG	mutant genotyping	
	LBb1.3	ATTTTGCCGATTTCGGAAC	mutant genotyping	
	EPFL8 2kb TOPO-XhoI-F	AAAGCAGGCTCCGCGGCCGCACTCGAGGAGAAATGGATCGATCTAAG	cloning	for pKUT12
	EPFL8 2kb TOPO-SpeI-R	GGCGCGCCCTCTAGAGGATCCACTAGTATCATCACAATTTTCTCAAA	cloning	for pKUT12
	EPFL8 cds Ass-F	TGAAGCTAGTCGACTCTAGCCTCGAGATGGATTCGTCAAGAAAATA	cloning	for pER8
	EPFL8 cds Ass-R	AAGCTGGGAGGCCTGGATCGACTAGTTTAAGGTTCAAATACTCTAT	cloning	for pER8
	EPFL8-F-qRT	GAGCTGCACTGTTCGTTGC	RT-qPCR	
	EPFL8-R-qRT	GTTCCGGCATTTTGTCGCAC	RT-qPCR	
**EPF1**	EPF1+207F_qRT	ATGCCGTCTTGTGATGGTTAG	RT-qPCR	
	EPF1+315rc_qRT	TCAAGGGACAGGGTAGGACTT	RT-qPCR	
**EPF2**	EPF2+60F_qRT	TTTGGTCGTTAACTCCATTCG	RT-qPCR	
	EPF2+225rc_qRT	ATCCGGTAAGCTTGATCCTGT	RT-qPCR	
**EPFL1**	EPFL1.1-F-qRT	CCTCCTATTTCTCCTCAAGTCGC	RT-qPCR	
	EPFL1.1-R-qRT	TAACTCGTGTGAAGCGGGAG	RT-qPCR	
**EPFL2**	EPFL2-F-qRT	AATGGTGTGGAGCAGCAACA	RT-qPCR	
	EPFL2-R-qRT	GTGAATTCGACAGAGTCAGGC	RT-qPCR	
**EPFL3**	EPFL3-F-qRT	GCAGGCCTATTGCTTCCAGT	RT-qPCR	
	EPFL3-R-qRT	TGCTTCACATGGCTCACATC	RT-qPCR	
**EPFL4**	EPFL4+231F_qRT	CCGGTTCACGTACCAATTCAA	RT-qPCR	
	EPFL4+298rc_qRT	ACCGCCAAGCTTCAGGGTAA	RT-qPCR	
**EPFL5**	EPFL5-F-qRT	CGTCCTCCCAACTCTCATCG	RT-qPCR	

	EPFL5-R-qRT	ATCTGACCCGGTAAACCCGA	RT-qPCR
**EPFL6**	EPFL6+487F_qRT	AGATCCGTGACCGGAGCAAG	RT-qPCR
	EPFL6+648rc_qRT	CGGGTAGTATTCGGCGGTGA	RT-qPCR
**EPFL7**	EPFL7.1-F-qRT	TTTGCGAGGTTGTAGATAAGGC	RT-qPCR
	EPFL7.1-R-qRT	CACTTGTAGACGAGAGGGCA	RT-qPCR
**EPFL9**	EPFL9-F-qRT	AATACGGTCTCCCTTCTCCCA	RT-qPCR
	EPFL9-R-qRT	TCGACTGGAACTTGCTCTGC	RT-qPCR
**ER**	ER-F-qRT	GACTTGTGATCCTTCTCATGGT	RT-qPCR
	ER-R-qRT	CGAGCTTCGGTGTCGAATAA	RT-qPCR
**ERL1**	ERL1-F-qRT	TGGATCACTTTGGGACCTTC	RT-qPCR
	ERL1-R-qRT	GGCTAGTCCTTGTGCAGCTC	RT-qPCR
**ERL2**	ERL2-F-qRT	GCGCAAGGACTTGCATATCT	RT-qPCR
	ERL2-R-qRT	TCGAAATTCCCATCAAGGAG	RT-qPCR
**TMM**	TMM-F-qRT	AAAACGCGTTCAAAGGGCTC	RT-qPCR
	TMM-R-qRT	GGTGAAGGACCCGAAGACTG	RT-qPCR
ACTIN2	ACTIN2 qRT-F	GATGAGGCAGGTCCAGGAATC	RT-qPCR
	ACTIN2 qRT-R	AACCCCAGCTTTTTAAGCCTTT	RT-qPCR
